# Supplementary material for: Bacterial genome-wide association study of hyper-virulent pneumococcal serotype 1 identifies genetic variation associated with neurotropism
Source: Commun Biol. 2020 Oct 8;3:559. doi: 10.1038/s42003-020-01290-9 (PMC7545184; doi:10.1038/s42003-020-01290-9)
Supplement: Supplementary file 1 — Supplementary Information [file 42003_2020_1290_MOESM1_ESM.pdf]

Bacterial genome-wide association study of hyper-virulent pneumococcal serotype 1 identifies genetic variation associated with neurotropism

Chrispin Chaguza, Marie Yang, Jennifer E. Cornick, Mignon du Plessis, Rebecca A. Gladstone, Brenda A. Kwambana-Adams, Stephanie W. Lo, Chinelo Ebruke, Gerry Tonkin-Hill, Chikondi Peno, Madikay Senghore, Stephen K. Obaro, Sani Ousmane, Gerd Pluschke, Jean-Marc Collard, Betuel Sigaùque, Neil French, Keith P. Klugman, Robert S. Heyderman, Lesley McGee, Martin Antonio, Robert F. Breiman, Anne von Gottberg, Dean B. Everett, Aras Kadioglu and Stephen D. Bentley

Other supplementary materials for this manuscript include the following:

Supplementary Data 1 (separate file): Summary of the pneumococcal serotype 1 isolates used in this study.

Supplementary Data 2 (separate file): Source data for the main text figures.

Supplementary Data 3 (separate file): Multiple sequence alignment showing sequence conservation of the genomic region containing the unitig ID 8805 in *pspC* gene.

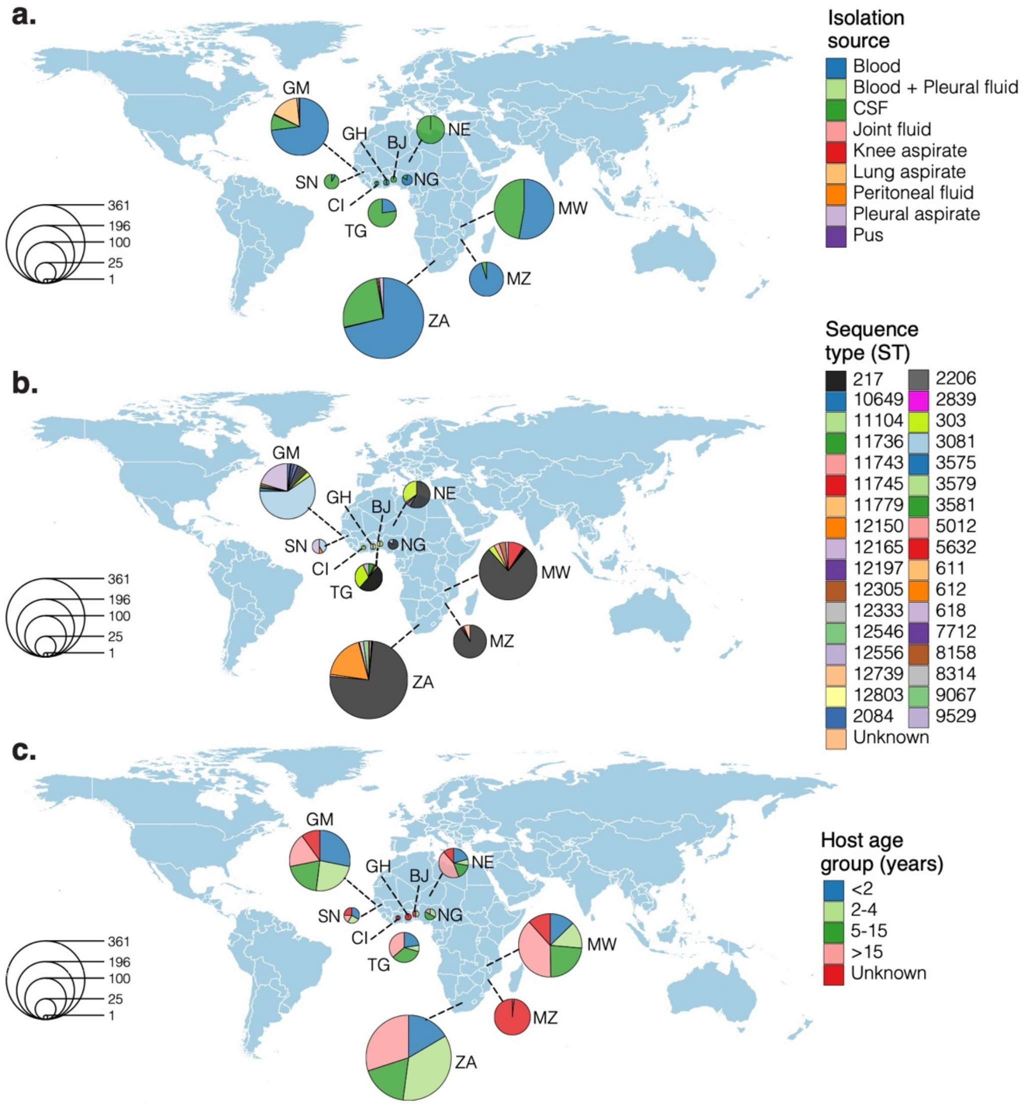

Supplementary Fig. 1. Characteristics of the African *S. pneumoniae* serotype 1 isolates by country of origin. The frequency of the isolates from each country by **a)** body isolation source, **b)** sequence type (ST) and **c)** host age (years) are shown as pie charts. The size of the pie charts is proportional to the number of isolates from each country as shown by the scale represented by the concentric circles at the bottom left of the diagram. The country names are designated by their international two letter codes as follows: South Africa (ZA), Malawi (MW), The Gambia (GM), Ghana (GH), Niger (NE), Nigeria (NG), Togo (TG), Benin (BJ), Côte d'Ivoire or Ivory Coast (CI) and Senegal (SN).

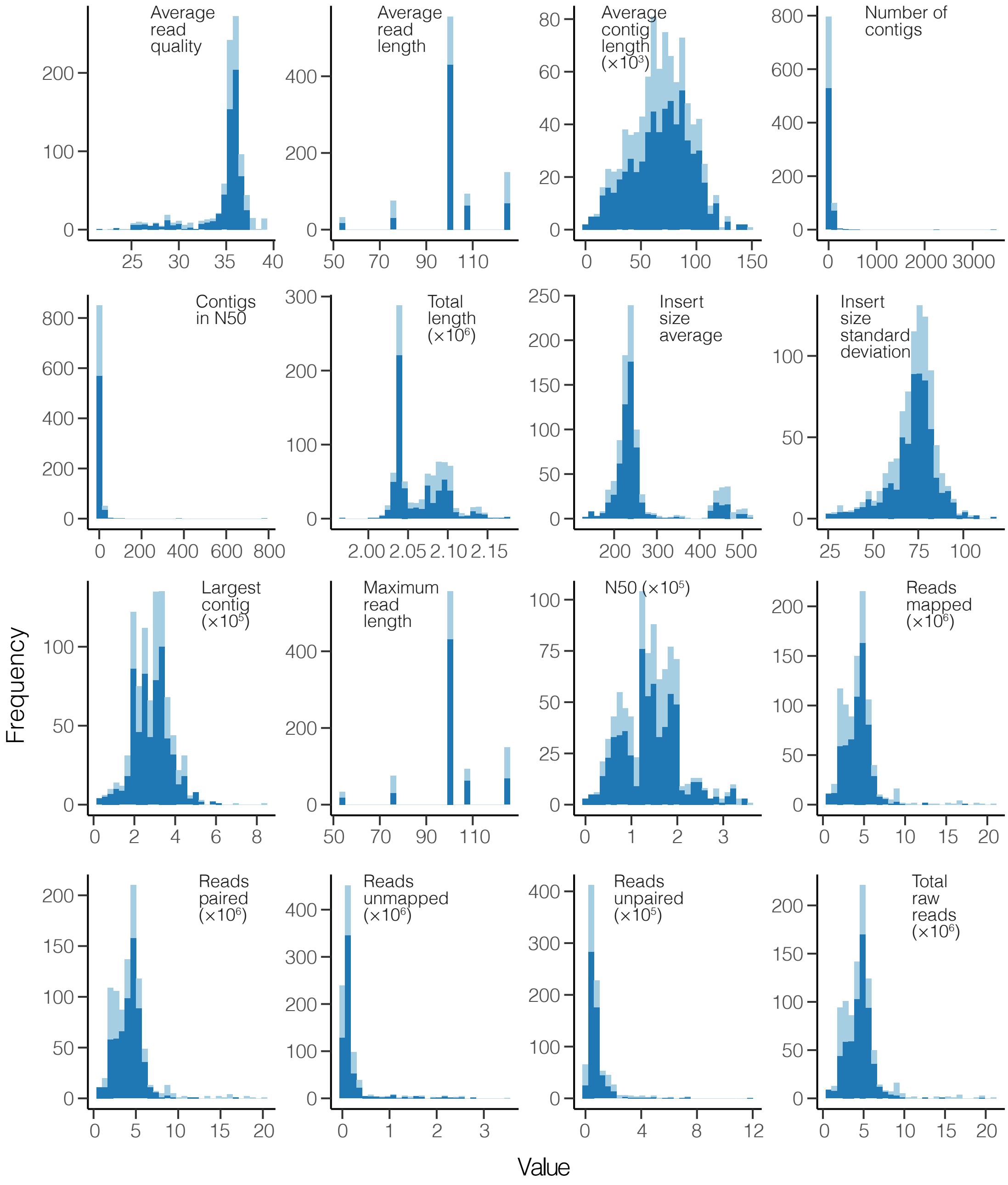

Supplementary Fig. 2. Summary of the assembly and mapping qualities for the whole genome sequencing data of the African *S. pneumoniae* serotype 1 isolates. The light- and dark blue colours corresponds to isolates sampled from cerebrospinal fluid (CSF) and non-CSF tissue respectively.

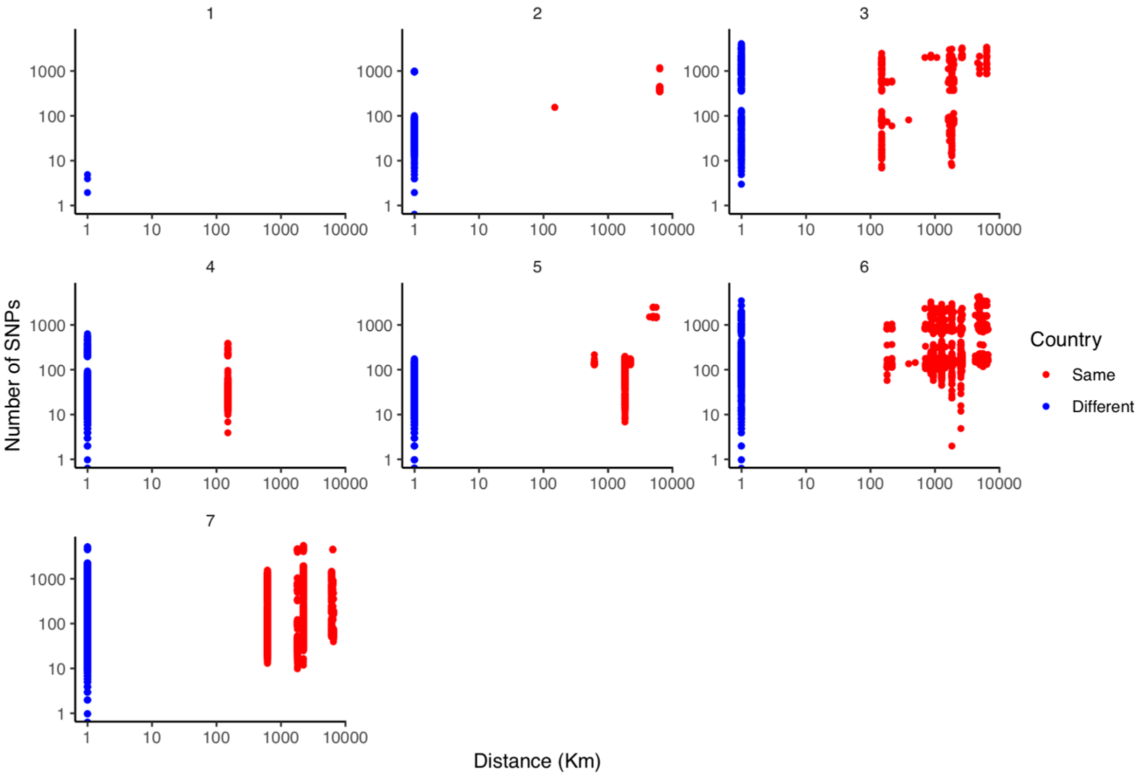

Supplementary Fig. 3. Relationship between genetic similarity and geographical proximity of the serotype 1 isolates in each clade. The scatter plots show the number of SNPs and geographical distance (in kilometers [Km]) for each pair of isolates. Both axes are shown in logarithmic scale (base 10) for clarity. The points in each plot are country by whether or not the isolates were sampled from the same country as shown in the key to the far right of the figure. The clade number is shown at the top of each diagram.

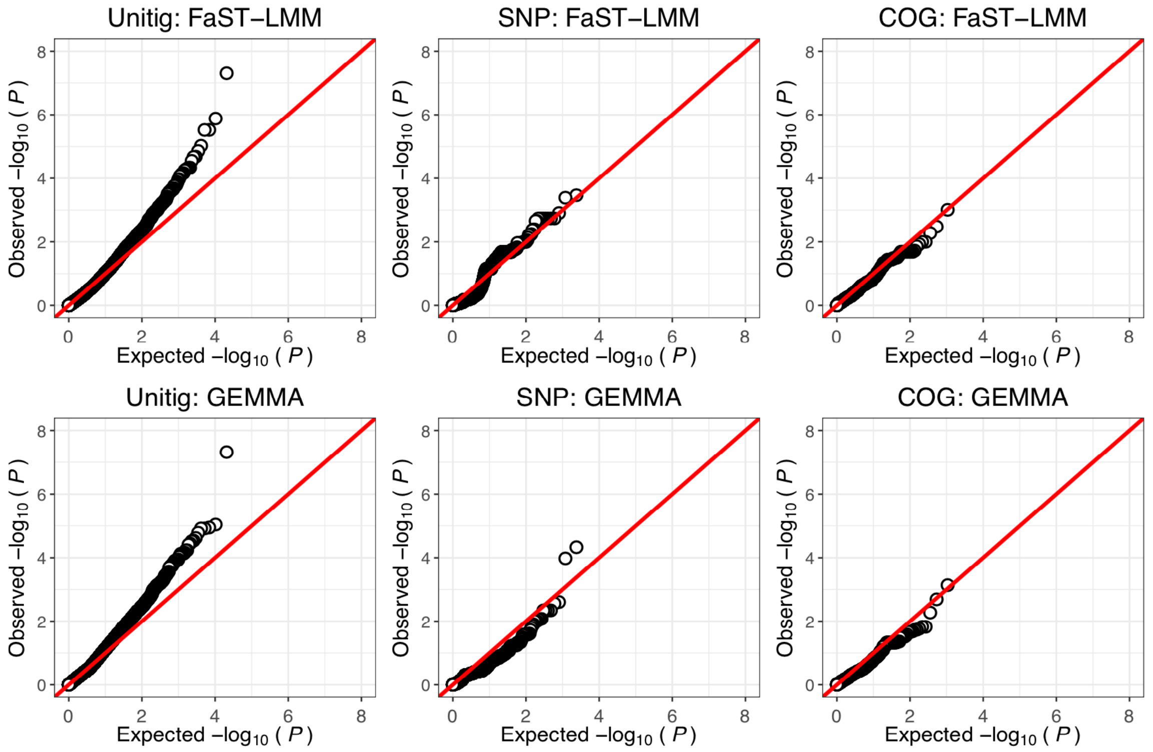

Supplementary Fig. 4. QQ-plots showing the expected and observed P-values for different GWAS analyses. The observed and expected P-values from the GWAS analysis of the SNPs, COGs and unitigs using GEMMA and FaST-LMM.

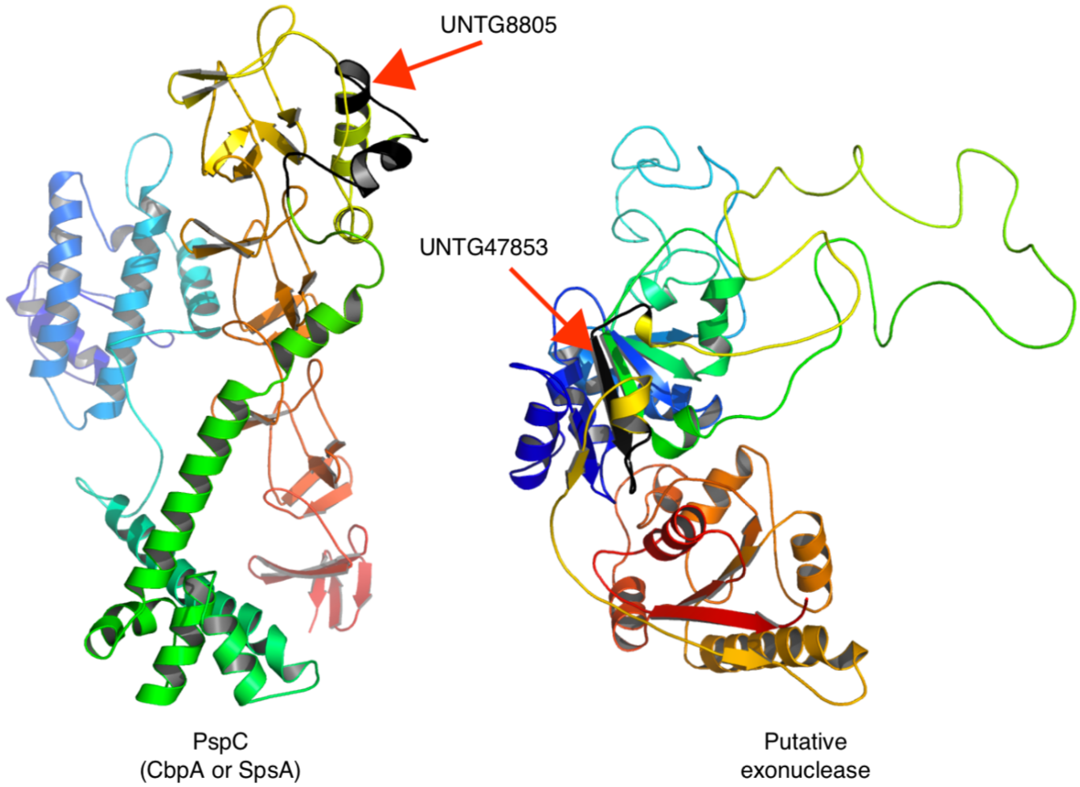

Supplementary Fig. 5. Predicted protein structure of the pneumococcal surface protein C (PspC*)*, also known as choline binding protein A (CbpA) and *spsA,* and a putative exonuclease. The full-chain protein structure of PspC was predicted using comparative and *de novo* methods implemented in Robetta server (https://robetta.bakerlab.org/) as no template sequence with sufficient coverage was found using the Swiss-Model automated protein structure homology-modelling server (https://swissmodel.expasy.org/) while the putative exonuclease was modelled using Swiss-Model. The location of the UNTG8805 unitig sequence in the α-helix proline-rich repeat region of PspC, and location of the unitig sequence UNTG47853 in the putative exonuclease within the β-sheet are shown in black colour.

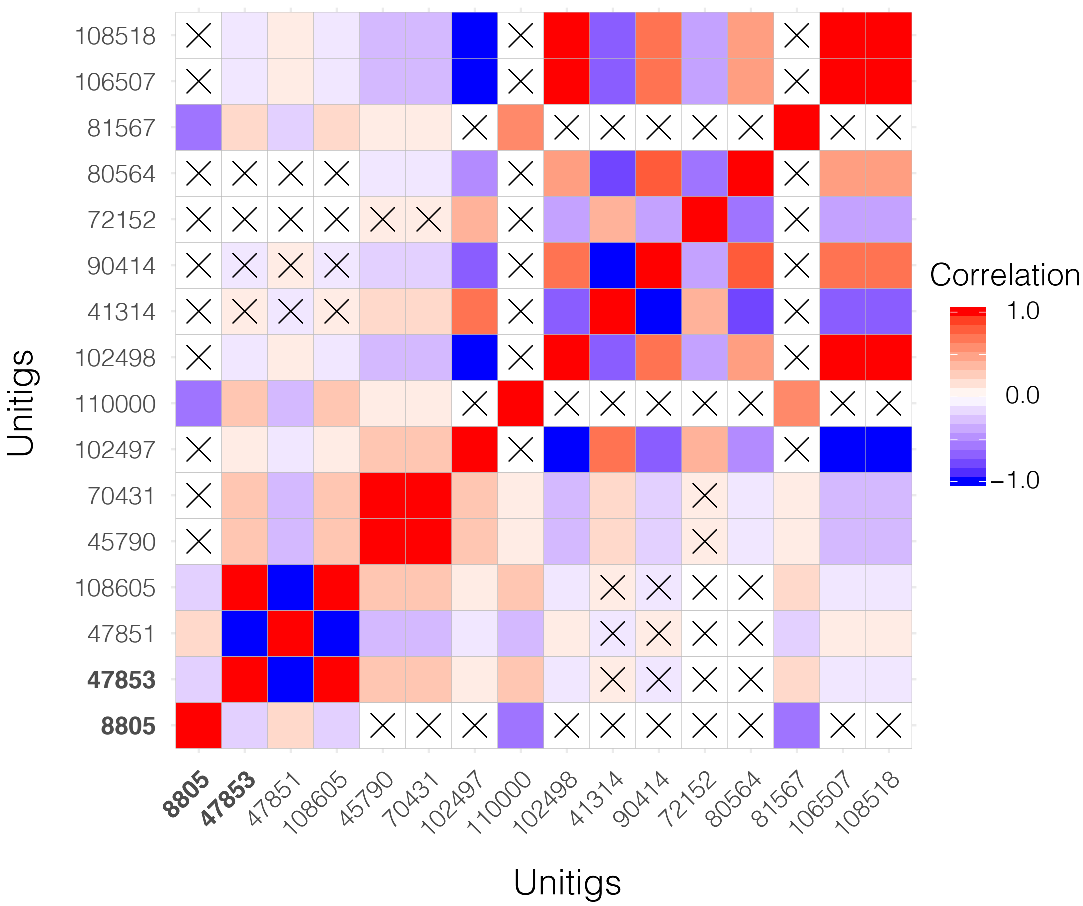

Supplementary Fig. 6. Correlation plots for the genome-wide significant and suggestive unitigs. The genome-wide significant unitigs are labelled in bold characters. The crosses show non-statistically significant correlation values with *P*-value >0.05.

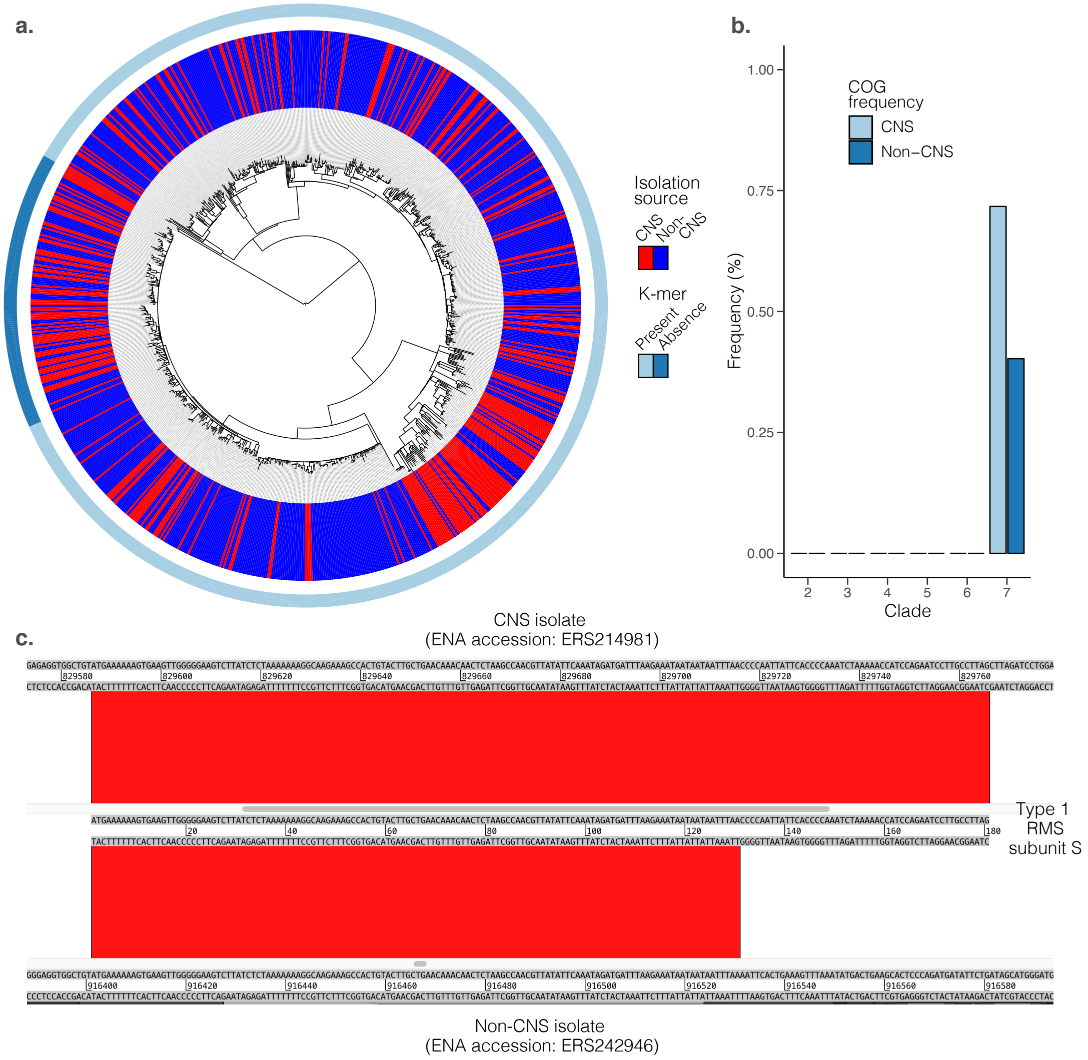

Supplementary Fig. 7. Differential phylogenetic and geographical distribution of the suggestive accessory gene. The top right panel represents the distribution of the genome-wide significant COG (ID 445) while the other panels show frequency of the suggestive COGs. Only lead unitigs with unique presence/absence patterns are shown in the figures.

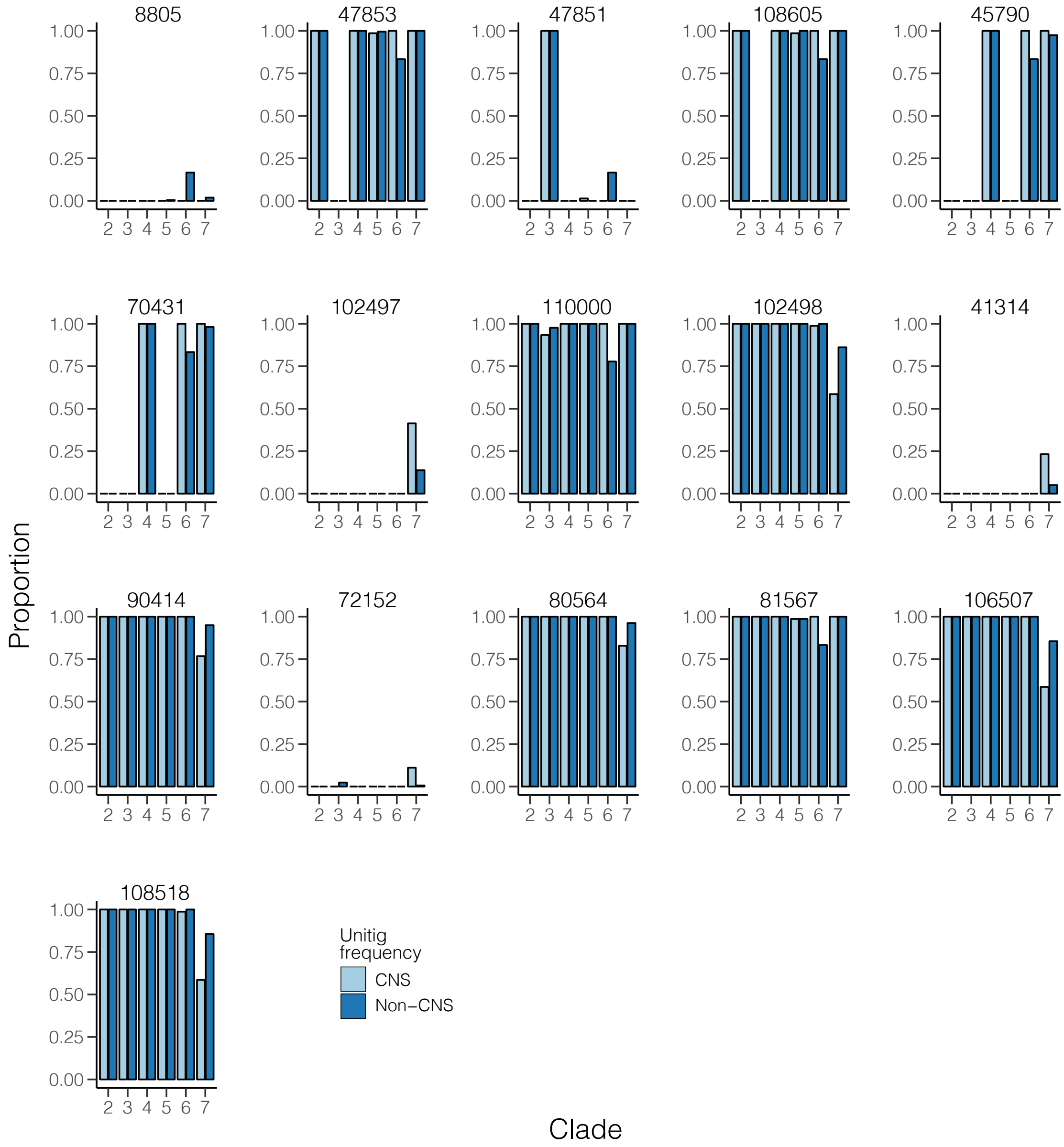

Supplementary Fig. 8. Phylogenetic and geographical distribution of the genome-wide significant and suggestive unitigs. The frequency of unitigs in CNS and non-CNS isolates in clades with >5 isolates.

Supplementary Table 1. Reference genome sequences used to annotate unitigs.

Accession ID	Strain ID	Genbank URL
NC_014498	670-6B	https://www.ncbi.nlm.nih.gov/nuccore/NC_014498
AE005672	TIGR4	https://www.ncbi.nlm.nih.gov/nuccore/AE005672
AKBW01000001	TIGR4	https://www.ncbi.nlm.nih.gov/nuccore/AKBW01000001
AP017971	KK0981	https://www.ncbi.nlm.nih.gov/nuccore/AP017971
AP018043	KK0381	https://www.ncbi.nlm.nih.gov/nuccore/AP018043
AP018044	KK1157	https://www.ncbi.nlm.nih.gov/nuccore/AP018044
AP018936	NU83127	https://www.ncbi.nlm.nih.gov/nuccore/AP018936
AP019192	ASP0581	https://www.ncbi.nlm.nih.gov/nuccore/AP019192
NC_014494	AP200	https://www.ncbi.nlm.nih.gov/nuccore/NC_014494
NC_011900	ATCC 700669	https://www.ncbi.nlm.nih.gov/nuccore/NC_011900
CP000918	70585	https://www.ncbi.nlm.nih.gov/nuccore/CP000918
CP000919	JJA	https://www.ncbi.nlm.nih.gov/nuccore/CP000919
CP000920	P1031	https://www.ncbi.nlm.nih.gov/nuccore/CP000920
CP000921	Taiwan19F-14	https://www.ncbi.nlm.nih.gov/nuccore/CP000921
CP000936	Hungary19A-6	https://www.ncbi.nlm.nih.gov/nuccore/CP000936
CP001033	CGSP14	https://www.ncbi.nlm.nih.gov/nuccore/CP001033
CP001845	gamPNI0373	https://www.ncbi.nlm.nih.gov/nuccore/CP001845
CP002121	AP200	https://www.ncbi.nlm.nih.gov/nuccore/CP002121
CP002176	670-6B	https://www.ncbi.nlm.nih.gov/nuccore/CP002176
CP003357	ST556	https://www.ncbi.nlm.nih.gov/nuccore/CP003357
CP007593	NT_110_58	https://www.ncbi.nlm.nih.gov/nuccore/CP007593
CP018136	SP49	https://www.ncbi.nlm.nih.gov/nuccore/CP018136
CP025076	strain 19F	https://www.ncbi.nlm.nih.gov/nuccore/CP025076
CP025256	Xen35	https://www.ncbi.nlm.nih.gov/nuccore/CP025256
CP026670	335	https://www.ncbi.nlm.nih.gov/nuccore/CP026670
CP031246	M26368	https://www.ncbi.nlm.nih.gov/nuccore/CP031246
CP031247	M23734	https://www.ncbi.nlm.nih.gov/nuccore/CP031247
CP031248	M26365	https://www.ncbi.nlm.nih.gov/nuccore/CP031248
CP035897	EF3030	https://www.ncbi.nlm.nih.gov/nuccore/CP035897
NC_008533	D39	https://www.ncbi.nlm.nih.gov/nuccore/NC_008533
FM211187	ATCC 700669	https://www.ncbi.nlm.nih.gov/nuccore/FM211187
NC_011072	G54	https://www.ncbi.nlm.nih.gov/nuccore/NC_011072
HE983624	SPNA45	https://www.ncbi.nlm.nih.gov/nuccore/HE983624
NC_010380	Hungary19A-6	https://www.ncbi.nlm.nih.gov/nuccore/NC_010380
NC_017592	OXC141	https://www.ncbi.nlm.nih.gov/nuccore/NC_017592
NC_003098	R6	https://www.ncbi.nlm.nih.gov/nuccore/NC_003098

CR931639	2616/39	https://www.ncbi.nlm.nih.gov/nuccore/CR931639
NC_017769	ST556	https://www.ncbi.nlm.nih.gov/nuccore/NC_017769
NC_014251	TCH8431/19A	https://www.ncbi.nlm.nih.gov/nuccore/NC_014251
NC_003028	TIGR4	https://www.ncbi.nlm.nih.gov/nuccore/NC_003028
NC_012469	Taiwan19F-14	https://www.ncbi.nlm.nih.gov/nuccore/NC_012469

Supplementary Table 2. Complete reference genomes used for annotation of variants.

Unitig ID	Unitig sequence
**8805**	AACCAGAAAAACCAGCTCCAAAACCAGAAAAACCAGCTGAA
**47853**	AGAAACCCTCTGACTAATCTCAAGAGTAGCTGATACTCCCAAGACTTGGCAACT
41314	CTTACGCAAGCCTTCTGGATAATCTACCAAAATTCCTAAGCCTTCTGCACTTGGACGAGGA
72152	AAATGTGGGCATAGAAAAAACGCCAGCTCACATGAGAA
80564	TTGACTCTCAATCATGGAAGCCAACCCCCTTCTCCAAAATGGAGCCAGCAAGAGT
81567	GTTCGGGTGTTATTGCCTTTAACCTAGGTGATCTCCATCCTCACGATCTTGCGACG
90414	CTTACGCAAGCCTTCTGGATAATCTACCAAGATTCCTAAGCCTTCTGCACTTGGACGAGGA
102497	GACACCACTTTTGGTCAGAGGGGTGCTGAGACTATCTGCTAACTGCTGGATAGAGTAGTCT
102498	GACACCACTTTTGGTCAGAGGGGTGCTGAGGCTATCTGC
106507	AGACTACTCTATCCAGCAGTTAGCAGATAGCCTCAG
108518	CTCTATCCAGCAGTTAGCAGATAGCCTCAGCACCCCTCTGACCAAA
110000	TTTCTGTAGCTGGTGTTGGACCTGTCGGATGCACTGGA
47853	AGAAACCCTCTGACTAATCTCAAGAGTAGCTGATACTCCCAAGACTTGGCAACT
47851	AGAAACCCTCTGACTAATCTCAAGAGTAGCCGATACTCCCAAGACTTGGCAACTCTCAGGA
108605	TCCTGAGAGTTGCCAAGTCTTGGGAGTATCAGCTACT
45790	TCTGCTAAACATTTTTTGGCATCCTCTATCACCTGCATGATG
70431	AAATGAAGTAGATGCCATCATGCAGGTGATAGAGGATGCCAAAAA
102497	GACACCACTTTTGGTCAGAGGGGTGCTGAGACTATCTGCTAACTGCTGGATAGAGTAGTCT
110000	TTTCTGTAGCTGGTGTTGGACCTGTCGGATGCACTGGA

The genome-wide significant unitigs are labelled in bold characters.
