# Supplementary material for: Bacterial genome-wide association study of hyper-virulent pneumococcal serotype 1 identifies genetic variation associated with neurotropism
Source: Commun Biol. 2020 Oct 8;3:559. doi: 10.1038/s42003-020-01290-9 (PMC7545184; doi:10.1038/s42003-020-01290-9)
Supplement: Supplementary file 2 — Description of Additional Supplementary Files [file 42003_2020_1290_MOESM2_ESM.pdf]

Description of Additional Supplementary Files

File Name: Supplementary Data 1
Description: Summary of the pneumococcal serotype 1 isolates used in this study

File Name: Supplementary Data 2
Description: Source data for the main text figures.

File Name: Supplementary Data 3
Description: Multiple sequence alignment showing sequence conservation of the genomic region containing the unitig ID 8805 in pspC gene.
