# Supplementary material for: Bacterial genome-wide association study of hyper-virulent pneumococcal serotype 1 identifies genetic variation associated with neurotropism
Source: Commun Biol. 2020 Oct 8;3:559. doi: 10.1038/s42003-020-01290-9 (PMC7545184; doi:10.1038/s42003-020-01290-9)
Supplement: Supplementary file 5 — Supplementary Data 3 [file 42003_2020_1290_MOESM5_ESM.pdf]

```
14410_5#43   AACCAGAAAAACCAGCTCCAAAACCAGAGAATCCAGCT---
14410_5#44   AACCAGAAAAACCAGCTCCAAAACCAGAGAATCCAGCT---
17175_7#39   AACCAGAAAAACCAGCTCCAAAACCAGAGAATCCAGCT---
17175_7#90   AACCAGAAAAACCAGCTCCAAAACCAGAGAATCCAGCT---
19341_1#2    AACCAGAAAAACCAGCTCCAAAACCAGAGAATCCAGCT---
20402_4#2    AACCAGAAAAACCAGCTCCAAAACCAGAGAATCCAGCT---
3850_2#7     AACCAGAAAAACCAGCTCCAAAACCAGAGAATCCAGCT---
4353_1#6     AACCAGAAAAACCAGCTCCAAAACCAGAGAATCCAGCT---
4525_1#3     AACCAGAAAAACCAGCTCCAAAACCAGAGAATCCAGCT---
6308_7#12    AACCAGAAAAACCAGCTCCAAAACCAGAGAATCCAGCT---
8786_8#27    AACCAGAAAAACCAGCTCCAAAACCAGAGAATCCAGCT---
10395_5#11   AACCAGAGAATCCAGCTCCAAAACCAGAAAAACCAGCTGAA
10395_5#30   AACCAGAGAATCCAGCTCCAAAACCAGAAAAACCAGCTGAA
10395_5#62   AACCAGAGAATCCAGCTCCAAAACCAGAAAAACCAGCTGAA
10395_5#7    AACCAGAGAATCCAGCTCCAAAACCAGAAAAACCAGCTGAA
10395_5#9    AACCAGAGAATCCAGCTCCAAAACCAGAAAAACCAGCTGAA
11658_1#58   AACCAGAGAATCCAGCTCCAAAACCAGAAAAACCAGCTGAA
11658_1#66   AACCAGAGAATCCAGCTCCAAAACCAGAAAAACCAGCTGAA
11658_1#69   AACCAGAGAATCCAGCTCCAAAACCAGAAAAACCAGCTGAA
11658_1#80   AACCAGAGAATCCAGCTCCAAAACCAGAAAAACCAGCTGAA
11658_1#88   AACCAGAGAATCCAGCTCCAAAACCAGAAAAACCAGCTGAA
11791_1#27   AACCAGAGAATCCAGCTCCAAAACCAGAAAAACCAGCTGAA
11791_1#38   AACCAGAGAATCCAGCTCCAAAACCAGAAAAACCAGCTGAA
11791_1#40   AACCAGAGAATCCAGCTCCAAAACCAGAAAAACCAGCTGAA
11791_1#51   AACCAGAGAATCCAGCTCCAAAACCAGAAAAACCAGCTGAA
12162_1#7    AACCAGAGAATCCAGCTCCAAAACCAGAAAAACCAGCTGAA
12290_2#53   AACCAGAGAATCCAGCTCCAAAACCAGAAAAACCAGCTGAA
12290_3#21   AACCAGAGAATCCAGCTCCAAAACCAGAAAAACCAGCTGAA
12290_3#55   AACCAGAGAATCCAGCTCCAAAACCAGAAAAACCAGCTGAA
12290_3#88   AACCAGAGAATCCAGCTCCAAAACCAGAAAAACCAGCTGAA
12290_3#89   AACCAGAGAATCCAGCTCCAAAACCAGAAAAACCAGCTGAA
12291_6#87   AACCAGAGAATCCAGCTCCAAAACCAGAAAAACCAGCTGAA
12378_2#1    AACCAGAGAATCCAGCTCCAAAACCAGAAAAACCAGCTGAA
12378_2#48   AACCAGAGAATCCAGCTCCAAAACCAGAAAAACCAGCTGAA
12837_1#14   AACCAGAGAATCCAGCTCCAAAACCAGAAAAACCAGCTGAA
12837_1#15   AACCAGAGAATCCAGCTCCAAAACCAGAAAAACCAGCTGAA
12837_1#16   AACCAGAGAATCCAGCTCCAAAACCAGAAAAACCAGCTGAA
12837_1#24   AACCAGAGAATCCAGCTCCAAAACCAGAAAAACCAGCTGAA
12837_1#37   AACCAGAGAATCCAGCTCCAAAACCAGAAAAACCAGCTGAA
12837_1#40   AACCAGAGAATCCAGCTCCAAAACCAGAAAAACCAGCTGAA
12837_1#51   AACCAGAGAATCCAGCTCCAAAACCAGAAAAACCAGCTGAA
12837_1#6    AACCAGAGAATCCAGCTCCAAAACCAGAAAAACCAGCTGAA
12837_1#74   AACCAGAGAATCCAGCTCCAAAACCAGAAAAACCAGCTGAA
12837_1#86   AACCAGAGAATCCAGCTCCAAAACCAGAAAAACCAGCTGAA
12837_2#18   AACCAGAGAATCCAGCTCCAAAACCAGAAAAACCAGCTGAA
12837_2#19   AACCAGAGAATCCAGCTCCAAAACCAGAAAAACCAGCTGAA
12837_2#22   AACCAGAGAATCCAGCTCCAAAACCAGAAAAACCAGCTGAA
12837_2#35   AACCAGAGAATCCAGCTCCAAAACCAGAAAAACCAGCTGAA
12837_2#42   AACCAGAGAATCCAGCTCCAAAACCAGAAAAACCAGCTGAA
12837_2#46   AACCAGAGAATCCAGCTCCAAAACCAGAAAAACCAGCTGAA
12837_2#51   AACCAGAGAATCCAGCTCCAAAACCAGAAAAACCAGCTGAA
12837_2#66   AACCAGAGAATCCAGCTCCAAAACCAGAAAAACCAGCTGAA
12837_2#69   AACCAGAGAATCCAGCTCCAAAACCAGAAAAACCAGCTGAA
12837_2#76   AACCAGAGAATCCAGCTCCAAAACCAGAAAAACCAGCTGAA
12837_2#84   AACCAGAGAATCCAGCTCCAAAACCAGAAAAACCAGCTGAA
12837_3#20   AACCAGAGAATCCAGCTCCAAAACCAGAAAAACCAGCTGAA
12837_3#27   AACCAGAGAATCCAGCTCCAAAACCAGAAAAACCAGCTGAA
12837_3#28   AACCAGAGAATCCAGCTCCAAAACCAGAAAAACCAGCTGAA
12837_3#54   AACCAGAGAATCCAGCTCCAAAACCAGAAAAACCAGCTGAA
12837_3#58   AACCAGAGAATCCAGCTCCAAAACCAGAAAAACCAGCTGAA
12837_3#62   AACCAGAGAATCCAGCTCCAAAACCAGAAAAACCAGCTGAA
12837_3#66   AACCAGAGAATCCAGCTCCAAAACCAGAAAAACCAGCTGAA
12837_3#69   AACCAGAGAATCCAGCTCCAAAACCAGAAAAACCAGCTGAA
12837_3#78   AACCAGAGAATCCAGCTCCAAAACCAGAAAAACCAGCTGAA
12837_4#10   AACCAGAGAATCCAGCTCCAAAACCAGAAAAACCAGCTGAA
12837_4#29   AACCAGAGAATCCAGCTCCAAAACCAGAAAAACCAGCTGAA
12837_4#32   AACCAGAGAATCCAGCTCCAAAACCAGAAAAACCAGCTGAA
12837_4#61   AACCAGAGAATCCAGCTCCAAAACCAGAAAAACCAGCTGAA
12837_4#63   AACCAGAGAATCCAGCTCCAAAACCAGAAAAACCAGCTGAA
12837_5#18   AACCAGAGAATCCAGCTCCAAAACCAGAAAAACCAGCTGAA
12837_5#25   AACCAGAGAATCCAGCTCCAAAACCAGAAAAACCAGCTGAA
12837_5#29   AACCAGAGAATCCAGCTCCAAAACCAGAAAAACCAGCTGAA
12837_5#36   AACCAGAGAATCCAGCTCCAAAACCAGAAAAACCAGCTGAA
12837_5#48   AACCAGAGAATCCAGCTCCAAAACCAGAAAAACCAGCTGAA
12837_5#53   AACCAGAGAATCCAGCTCCAAAACCAGAAAAACCAGCTGAA
```

12837_5#77	AACCAGAGAATCCAGCTCCAAAACCAGAAAAACCAGCTGAA
12837_5#9	AACCAGAGAATCCAGCTCCAAAACCAGAAAAACCAGCTGAA
13154_4#10	AACCAGAGAATCCAGCTCCAAAACCAGAAAAACCAGCTGAA
13154_4#7	AACCAGAGAATCCAGCTCCAAAACCAGAAAAACCAGCTGAA
13414_8#1	AACCAGAGAATCCAGCTCCAAAACCAGAAAAACCAGCTGAA
13414_8#15	AACCAGAGAATCCAGCTCCAAAACCAGAAAAACCAGCTGAA
13414_8#18	AACCAGAGAATCCAGCTCCAAAACCAGAAAAACCAGCTGAA
13414_8#25	AACCAGAGAATCCAGCTCCAAAACCAGAAAAACCAGCTGAA
13414_8#29	AACCAGAGAATCCAGCTCCAAAACCAGAAAAACCAGCTGAA
13414_8#30	AACCAGAGAATCCAGCTCCAAAACCAGAAAAACCAGCTGAA
13414_8#36	AACCAGAGAATCCAGCTCCAAAACCAGAAAAACCAGCTGAA
13414_8#47	AACCAGAGAATCCAGCTCCAAAACCAGAAAAACCAGCTGAA
13414_8#54	AACCAGAGAATCCAGCTCCAAAACCAGAAAAACCAGCTGAA
13414_8#62	AACCAGAGAATCCAGCTCCAAAACCAGAAAAACCAGCTGAA
13414_8#73	AACCAGAGAATCCAGCTCCAAAACCAGAAAAACCAGCTGAA
13414_8#76	AACCAGAGAATCCAGCTCCAAAACCAGAAAAACCAGCTGAA
13415_4#12	AACCAGAGAATCCAGCTCCAAAACCAGAAAAACCAGCTGAA
13415_4#15	AACCAGAGAATCCAGCTCCAAAACCAGAAAAACCAGCTGAA
13415_4#23	AACCAGAGAATCCAGCTCCAAAACCAGAAAAACCAGCTGAA
13415_4#31	AACCAGAGAATCCAGCTCCAAAACCAGAAAAACCAGCTGAA
13415_4#33	AACCAGAGAATCCAGCTCCAAAACCAGAAAAACCAGCTGAA
13415_4#36	AACCAGAGAATCCAGCTCCAAAACCAGAAAAACCAGCTGAA
13415_4#4	AACCAGAGAATCCAGCTCCAAAACCAGAAAAACCAGCTGAA
13415_4#47	AACCAGAGAATCCAGCTCCAAAACCAGAAAAACCAGCTGAA
13415_4#52	AACCAGAGAATCCAGCTCCAAAACCAGAAAAACCAGCTGAA
13415_4#68	AACCAGAGAATCCAGCTCCAAAACCAGAAAAACCAGCTGAA
13415_4#69	AACCAGAGAATCCAGCTCCAAAACCAGAAAAACCAGCTGAA
13415_4#7	AACCAGAGAATCCAGCTCCAAAACCAGAAAAACCAGCTGAA
13415_4#85	AACCAGAGAATCCAGCTCCAAAACCAGAAAAACCAGCTGAA
13415_4#87	AACCAGAGAATCCAGCTCCAAAACCAGAAAAACCAGCTGAA
13415_4#88	AACCAGAGAATCCAGCTCCAAAACCAGAAAAACCAGCTGAA
13415_5#20	AACCAGAGAATCCAGCTCCAAAACCAGAAAAACCAGCTGAA
13415_5#22	AACCAGAGAATCCAGCTCCAAAACCAGAAAAACCAGCTGAA
13415_5#27	AACCAGAGAATCCAGCTCCAAAACCAGAAAAACCAGCTGAA
13415_5#3	AACCAGAGAATCCAGCTCCAAAACCAGAAAAACCAGCTGAA
13415_5#37	AACCAGAGAATCCAGCTCCAAAACCAGAAAAACCAGCTGAA
13415_5#43	AACCAGAGAATCCAGCTCCAAAACCAGAAAAACCAGCTGAA
13415_5#60	AACCAGAGAATCCAGCTCCAAAACCAGAAAAACCAGCTGAA
13415_5#67	AACCAGAGAATCCAGCTCCAAAACCAGAAAAACCAGCTGAA
13415_5#68	AACCAGAGAATCCAGCTCCAAAACCAGAAAAACCAGCTGAA
13415_5#69	AACCAGAGAATCCAGCTCCAAAACCAGAAAAACCAGCTGAA
13415_5#73	AACCAGAGAATCCAGCTCCAAAACCAGAAAAACCAGCTGAA
13415_5#82	AACCAGAGAATCCAGCTCCAAAACCAGAAAAACCAGCTGAA
13415_5#89	AACCAGAGAATCCAGCTCCAAAACCAGAAAAACCAGCTGAA
13415_6#13	AACCAGAGAATCCAGCTCCAAAACCAGAAAAACCAGCTGAA
13415_6#26	AACCAGAGAATCCAGCTCCAAAACCAGAAAAACCAGCTGAA
13415_6#39	AACCAGAGAATCCAGCTCCAAAACCAGAAAAACCAGCTGAA
13415_6#4	AACCAGAGAATCCAGCTCCAAAACCAGAAAAACCAGCTGAA
13415_6#44	AACCAGAGAATCCAGCTCCAAAACCAGAAAAACCAGCTGAA
13415_6#50	AACCAGAGAATCCAGCTCCAAAACCAGAAAAACCAGCTGAA
13415_6#58	AACCAGAGAATCCAGCTCCAAAACCAGAAAAACCAGCTGAA
13415_6#62	AACCAGAGAATCCAGCTCCAAAACCAGAAAAACCAGCTGAA
13415_6#72	AACCAGAGAATCCAGCTCCAAAACCAGAAAAACCAGCTGAA
13415_6#81	AACCAGAGAATCCAGCTCCAAAACCAGAAAAACCAGCTGAA
13415_6#90	AACCAGAGAATCCAGCTCCAAAACCAGAAAAACCAGCTGAA
13415_6#93	AACCAGAGAATCCAGCTCCAAAACCAGAAAAACCAGCTGAA
13415_6#94	AACCAGAGAATCCAGCTCCAAAACCAGAAAAACCAGCTGAA
13415_6#95	AACCAGAGAATCCAGCTCCAAAACCAGAAAAACCAGCTGAA
13415_7#30	AACCAGAGAATCCAGCTCCAAAACCAGAAAAACCAGCTGAA
13415_7#31	AACCAGAGAATCCAGCTCCAAAACCAGAAAAACCAGCTGAA
13415_7#48	AACCAGAGAATCCAGCTCCAAAACCAGAAAAACCAGCTGAA
13415_7#50	AACCAGAGAATCCAGCTCCAAAACCAGAAAAACCAGCTGAA
13415_7#61	AACCAGAGAATCCAGCTCCAAAACCAGAAAAACCAGCTGAA
13415_7#62	AACCAGAGAATCCAGCTCCAAAACCAGAAAAACCAGCTGAA
13415_7#87	AACCAGAGAATCCAGCTCCAAAACCAGAAAAACCAGCTGAA
13415_8#14	AACCAGAGAATCCAGCTCCAAAACCAGAAAAACCAGCTGAA
13415_8#19	AACCAGAGAATCCAGCTCCAAAACCAGAAAAACCAGCTGAA
13415_8#25	AACCAGAGAATCCAGCTCCAAAACCAGAAAAACCAGCTGAA
13415_8#27	AACCAGAGAATCCAGCTCCAAAACCAGAAAAACCAGCTGAA
13415_8#37	AACCAGAGAATCCAGCTCCAAAACCAGAAAAACCAGCTGAA
13415_8#38	AACCAGAGAATCCAGCTCCAAAACCAGAAAAACCAGCTGAA
13415_8#40	AACCAGAGAATCCAGCTCCAAAACCAGAAAAACCAGCTGAA
13415_8#5	AACCAGAGAATCCAGCTCCAAAACCAGAAAAACCAGCTGAA
13415_8#51	AACCAGAGAATCCAGCTCCAAAACCAGAAAAACCAGCTGAA
13415_8#68	AACCAGAGAATCCAGCTCCAAAACCAGAAAAACCAGCTGAA
13415_8#74	AACCAGAGAATCCAGCTCCAAAACCAGAAAAACCAGCTGAA
13415_8#82	AACCAGAGAATCCAGCTCCAAAACCAGAAAAACCAGCTGAA
13415_8#91	AACCAGAGAATCCAGCTCCAAAACCAGAAAAACCAGCTGAA
13470_1#41	AACCAGAGAATCCAGCTCCAAAACCAGAAAAACCAGCTGAA

13470_1#46	AACCAGAGAATCCAGCTCCAAAACCAGAAAAACCAGCTGAA
13470_1#53	AACCAGAGAATCCAGCTCCAAAACCAGAAAAACCAGCTGAA
13470_1#69	AACCAGAGAATCCAGCTCCAAAACCAGAAAAACCAGCTGAA
13470_1#70	AACCAGAGAATCCAGCTCCAAAACCAGAAAAACCAGCTGAA
14257_1#1	AACCAGAGAATCCAGCTCCAAAACCAGAAAAACCAGCTGAA
14257_1#16	AACCAGAGAATCCAGCTCCAAAACCAGAAAAACCAGCTGAA
14257_1#32	AACCAGAGAATCCAGCTCCAAAACCAGAAAAACCAGCTGAA
14257_1#4	AACCAGAGAATCCAGCTCCAAAACCAGAAAAACCAGCTGAA
14257_1#44	AACCAGAGAATCCAGCTCCAAAACCAGAAAAACCAGCTGAA
14257_1#5	AACCAGAGAATCCAGCTCCAAAACCAGAAAAACCAGCTGAA
14257_1#60	AACCAGAGAATCCAGCTCCAAAACCAGAAAAACCAGCTGAA
14257_1#69	AACCAGAGAATCCAGCTCCAAAACCAGAAAAACCAGCTGAA
14257_1#72	AACCAGAGAATCCAGCTCCAAAACCAGAAAAACCAGCTGAA
14257_1#78	AACCAGAGAATCCAGCTCCAAAACCAGAAAAACCAGCTGAA
14257_1#83	AACCAGAGAATCCAGCTCCAAAACCAGAAAAACCAGCTGAA
14324_6#54	AACCAGAGAATCCAGCTCCAAAACCAGAAAAACCAGCTGAA
14410_4#1	AACCAGAGAATCCAGCTCCAAAACCAGAAAAACCAGCTGAA
14410_4#10	AACCAGAGAATCCAGCTCCAAAACCAGAAAAACCAGCTGAA
14410_4#11	AACCAGAGAATCCAGCTCCAAAACCAGAAAAACCAGCTGAA
14410_4#20	AACCAGAGAATCCAGCTCCAAAACCAGAAAAACCAGCTGAA
14410_4#4	AACCAGAGAATCCAGCTCCAAAACCAGAAAAACCAGCTGAA
14410_4#43	AACCAGAGAATCCAGCTCCAAAACCAGAAAAACCAGCTGAA
14410_4#52	AACCAGAGAATCCAGCTCCAAAACCAGAAAAACCAGCTGAA
14410_4#54	AACCAGAGAATCCAGCTCCAAAACCAGAAAAACCAGCTGAA
14410_4#56	AACCAGAGAATCCAGCTCCAAAACCAGAAAAACCAGCTGAA
14410_4#60	AACCAGAGAATCCAGCTCCAAAACCAGAAAAACCAGCTGAA
14410_4#63	AACCAGAGAATCCAGCTCCAAAACCAGAAAAACCAGCTGAA
14410_4#71	AACCAGAGAATCCAGCTCCAAAACCAGAAAAACCAGCTGAA
14410_4#81	AACCAGAGAATCCAGCTCCAAAACCAGAAAAACCAGCTGAA
14410_4#82	AACCAGAGAATCCAGCTCCAAAACCAGAAAAACCAGCTGAA
14410_4#85	AACCAGAGAATCCAGCTCCAAAACCAGAAAAACCAGCTGAA
14410_5#18	AACCAGAGAATCCAGCTCCAAAACCAGAAAAACCAGCTGAA
14410_5#21	AACCAGAGAATCCAGCTCCAAAACCAGAAAAACCAGCTGAA
14410_5#24	AACCAGAGAATCCAGCTCCAAAACCAGAAAAACCAGCTGAA
14410_5#35	AACCAGAGAATCCAGCTCCAAAACCAGAAAAACCAGCTGAA
14410_5#42	AACCAGAGAATCCAGCTCCAAAACCAGAAAAACCAGCTGAA
14410_5#45	AACCAGAGAATCCAGCTCCAAAACCAGAAAAACCAGCTGAA
14410_5#47	AACCAGAGAATCCAGCTCCAAAACCAGAAAAACCAGCTGAA
14410_5#48	AACCAGAGAATCCAGCTCCAAAACCAGAAAAACCAGCTGAA
14410_5#49	AACCAGAGAATCCAGCTCCAAAACCAGAAAAACCAGCTGAA
14410_5#50	AACCAGAGAATCCAGCTCCAAAACCAGAAAAACCAGCTGAA
14410_5#7	AACCAGAGAATCCAGCTCCAAAACCAGAAAAACCAGCTGAA
14410_6#1	AACCAGAGAATCCAGCTCCAAAACCAGAAAAACCAGCTGAA
14410_6#14	AACCAGAGAATCCAGCTCCAAAACCAGAAAAACCAGCTGAA
14410_6#19	AACCAGAGAATCCAGCTCCAAAACCAGAAAAACCAGCTGAA
14410_6#26	AACCAGAGAATCCAGCTCCAAAACCAGAAAAACCAGCTGAA
14410_6#33	AACCAGAGAATCCAGCTCCAAAACCAGAAAAACCAGCTGAA
14410_6#38	AACCAGAGAATCCAGCTCCAAAACCAGAAAAACCAGCTGAA
14410_6#39	AACCAGAGAATCCAGCTCCAAAACCAGAAAAACCAGCTGAA
14410_6#4	AACCAGAGAATCCAGCTCCAAAACCAGAAAAACCAGCTGAA
14410_6#41	AACCAGAGAATCCAGCTCCAAAACCAGAAAAACCAGCTGAA
14410_6#56	AACCAGAGAATCCAGCTCCAAAACCAGAAAAACCAGCTGAA
14410_6#64	AACCAGAGAATCCAGCTCCAAAACCAGAAAAACCAGCTGAA
14410_6#8	AACCAGAGAATCCAGCTCCAAAACCAGAAAAACCAGCTGAA
14410_6#82	AACCAGAGAATCCAGCTCCAAAACCAGAAAAACCAGCTGAA
14410_7#1	AACCAGAGAATCCAGCTCCAAAACCAGAAAAACCAGCTGAA
14410_7#11	AACCAGAGAATCCAGCTCCAAAACCAGAAAAACCAGCTGAA
14410_7#12	AACCAGAGAATCCAGCTCCAAAACCAGAAAAACCAGCTGAA
14410_7#14	AACCAGAGAATCCAGCTCCAAAACCAGAAAAACCAGCTGAA
14410_7#46	AACCAGAGAATCCAGCTCCAAAACCAGAAAAACCAGCTGAA
14410_7#52	AACCAGAGAATCCAGCTCCAAAACCAGAAAAACCAGCTGAA
14410_7#54	AACCAGAGAATCCAGCTCCAAAACCAGAAAAACCAGCTGAA
14410_7#61	AACCAGAGAATCCAGCTCCAAAACCAGAAAAACCAGCTGAA
14410_7#69	AACCAGAGAATCCAGCTCCAAAACCAGAAAAACCAGCTGAA
14410_7#76	AACCAGAGAATCCAGCTCCAAAACCAGAAAAACCAGCTGAA
14410_7#83	AACCAGAGAATCCAGCTCCAAAACCAGAAAAACCAGCTGAA
14410_7#9	AACCAGAGAATCCAGCTCCAAAACCAGAAAAACCAGCTGAA
14410_8#33	AACCAGAGAATCCAGCTCCAAAACCAGAAAAACCAGCTGAA
14410_8#49	AACCAGAGAATCCAGCTCCAAAACCAGAAAAACCAGCTGAA
14410_8#60	AACCAGAGAATCCAGCTCCAAAACCAGAAAAACCAGCTGAA
14410_8#67	AACCAGAGAATCCAGCTCCAAAACCAGAAAAACCAGCTGAA
14410_8#73	AACCAGAGAATCCAGCTCCAAAACCAGAAAAACCAGCTGAA
14412_1#16	AACCAGAGAATCCAGCTCCAAAACCAGAAAAACCAGCTGAA
14412_1#2	AACCAGAGAATCCAGCTCCAAAACCAGAAAAACCAGCTGAA
14412_1#31	AACCAGAGAATCCAGCTCCAAAACCAGAAAAACCAGCTGAA
14412_1#39	AACCAGAGAATCCAGCTCCAAAACCAGAAAAACCAGCTGAA
14412_1#43	AACCAGAGAATCCAGCTCCAAAACCAGAAAAACCAGCTGAA
14412_1#53	AACCAGAGAATCCAGCTCCAAAACCAGAAAAACCAGCTGAA
14412_1#54	AACCAGAGAATCCAGCTCCAAAACCAGAAAAACCAGCTGAA

14412_1#56	AACCAGAGAATCCAGCTCCAAAACCAGAAAAACCAGCTGAA
14412_1#70	AACCAGAGAATCCAGCTCCAAAACCAGAAAAACCAGCTGAA
14412_1#80	AACCAGAGAATCCAGCTCCAAAACCAGAAAAACCAGCTGAA
14412_1#83	AACCAGAGAATCCAGCTCCAAAACCAGAAAAACCAGCTGAA
14412_1#84	AACCAGAGAATCCAGCTCCAAAACCAGAAAAACCAGCTGAA
14412_1#88	AACCAGAGAATCCAGCTCCAAAACCAGAAAAACCAGCTGAA
14412_1#91	AACCAGAGAATCCAGCTCCAAAACCAGAAAAACCAGCTGAA
14412_1#93	AACCAGAGAATCCAGCTCCAAAACCAGAAAAACCAGCTGAA
14412_2#1	AACCAGAGAATCCAGCTCCAAAACCAGAAAAACCAGCTGAA
14412_2#16	AACCAGAGAATCCAGCTCCAAAACCAGAAAAACCAGCTGAA
14412_2#17	AACCAGAGAATCCAGCTCCAAAACCAGAAAAACCAGCTGAA
14412_2#2	AACCAGAGAATCCAGCTCCAAAACCAGAAAAACCAGCTGAA
14412_2#28	AACCAGAGAATCCAGCTCCAAAACCAGAAAAACCAGCTGAA
14412_2#3	AACCAGAGAATCCAGCTCCAAAACCAGAAAAACCAGCTGAA
14412_2#30	AACCAGAGAATCCAGCTCCAAAACCAGAAAAACCAGCTGAA
14412_2#31	AACCAGAGAATCCAGCTCCAAAACCAGAAAAACCAGCTGAA
14412_2#36	AACCAGAGAATCCAGCTCCAAAACCAGAAAAACCAGCTGAA
14412_2#4	AACCAGAGAATCCAGCTCCAAAACCAGAAAAACCAGCTGAA
14412_2#48	AACCAGAGAATCCAGCTCCAAAACCAGAAAAACCAGCTGAA
14412_2#62	AACCAGAGAATCCAGCTCCAAAACCAGAAAAACCAGCTGAA
14412_2#80	AACCAGAGAATCCAGCTCCAAAACCAGAAAAACCAGCTGAA
14520_6#58	AACCAGAGAATCCAGCTCCAAAACCAGAAAAACCAGCTGAA
14520_6#61	AACCAGAGAATCCAGCTCCAAAACCAGAAAAACCAGCTGAA
14520_6#63	AACCAGAGAATCCAGCTCCAAAACCAGAAAAACCAGCTGAA
14520_6#73	AACCAGAGAATCCAGCTCCAAAACCAGAAAAACCAGCTGAA
14520_6#79	AACCAGAGAATCCAGCTCCAAAACCAGAAAAACCAGCTGAA
14520_6#81	AACCAGAGAATCCAGCTCCAAAACCAGAAAAACCAGCTGAA
14520_6#94	AACCAGAGAATCCAGCTCCAAAACCAGAAAAACCAGCTGAA
14520_7#1	AACCAGAGAATCCAGCTCCAAAACCAGAAAAACCAGCTGAA
14520_7#5	AACCAGAGAATCCAGCTCCAAAACCAGAAAAACCAGCTGAA
14520_7#6	AACCAGAGAATCCAGCTCCAAAACCAGAAAAACCAGCTGAA
14520_7#77	AACCAGAGAATCCAGCTCCAAAACCAGAAAAACCAGCTGAA
14520_7#78	AACCAGAGAATCCAGCTCCAAAACCAGAAAAACCAGCTGAA
14520_7#79	AACCAGAGAATCCAGCTCCAAAACCAGAAAAACCAGCTGAA
14520_7#81	AACCAGAGAATCCAGCTCCAAAACCAGAAAAACCAGCTGAA
14520_7#84	AACCAGAGAATCCAGCTCCAAAACCAGAAAAACCAGCTGAA
14520_7#89	AACCAGAGAATCCAGCTCCAAAACCAGAAAAACCAGCTGAA
14555_3#20	AACCAGAGAATCCAGCTCCAAAACCAGAAAAACCAGCTGAA
14555_3#24	AACCAGAGAATCCAGCTCCAAAACCAGAAAAACCAGCTGAA
14555_3#25	AACCAGAGAATCCAGCTCCAAAACCAGAAAAACCAGCTGAA
14555_3#3	AACCAGAGAATCCAGCTCCAAAACCAGAAAAACCAGCTGAA
14555_3#34	AACCAGAGAATCCAGCTCCAAAACCAGAAAAACCAGCTGAA
14555_3#69	AACCAGAGAATCCAGCTCCAAAACCAGAAAAACCAGCTGAA
14555_3#74	AACCAGAGAATCCAGCTCCAAAACCAGAAAAACCAGCTGAA
14555_3#79	AACCAGAGAATCCAGCTCCAAAACCAGAAAAACCAGCTGAA
14555_3#90	AACCAGAGAATCCAGCTCCAAAACCAGAAAAACCAGCTGAA
14555_3#91	AACCAGAGAATCCAGCTCCAAAACCAGAAAAACCAGCTGAA
14555_4#1	AACCAGAGAATCCAGCTCCAAAACCAGAAAAACCAGCTGAA
14555_4#14	AACCAGAGAATCCAGCTCCAAAACCAGAAAAACCAGCTGAA
14555_4#47	AACCAGAGAATCCAGCTCCAAAACCAGAAAAACCAGCTGAA
14555_4#63	AACCAGAGAATCCAGCTCCAAAACCAGAAAAACCAGCTGAA
14555_4#79	AACCAGAGAATCCAGCTCCAAAACCAGAAAAACCAGCTGAA
14555_4#81	AACCAGAGAATCCAGCTCCAAAACCAGAAAAACCAGCTGAA
14555_4#89	AACCAGAGAATCCAGCTCCAAAACCAGAAAAACCAGCTGAA
14555_4#9	AACCAGAGAATCCAGCTCCAAAACCAGAAAAACCAGCTGAA
14566_4#1	AACCAGAGAATCCAGCTCCAAAACCAGAAAAACCAGCTGAA
14566_4#19	AACCAGAGAATCCAGCTCCAAAACCAGAAAAACCAGCTGAA
14566_4#22	AACCAGAGAATCCAGCTCCAAAACCAGAAAAACCAGCTGAA
14566_4#43	AACCAGAGAATCCAGCTCCAAAACCAGAAAAACCAGCTGAA
14566_4#44	AACCAGAGAATCCAGCTCCAAAACCAGAAAAACCAGCTGAA
14566_4#49	AACCAGAGAATCCAGCTCCAAAACCAGAAAAACCAGCTGAA
14566_4#63	AACCAGAGAATCCAGCTCCAAAACCAGAAAAACCAGCTGAA
14566_4#66	AACCAGAGAATCCAGCTCCAAAACCAGAAAAACCAGCTGAA
14566_4#67	AACCAGAGAATCCAGCTCCAAAACCAGAAAAACCAGCTGAA
14566_4#71	AACCAGAGAATCCAGCTCCAAAACCAGAAAAACCAGCTGAA
14566_4#73	AACCAGAGAATCCAGCTCCAAAACCAGAAAAACCAGCTGAA
14566_4#82	AACCAGAGAATCCAGCTCCAAAACCAGAAAAACCAGCTGAA
14566_4#86	AACCAGAGAATCCAGCTCCAAAACCAGAAAAACCAGCTGAA
14566_4#87	AACCAGAGAATCCAGCTCCAAAACCAGAAAAACCAGCTGAA
14566_5#18	AACCAGAGAATCCAGCTCCAAAACCAGAAAAACCAGCTGAA
14566_5#20	AACCAGAGAATCCAGCTCCAAAACCAGAAAAACCAGCTGAA
14566_5#66	AACCAGAGAATCCAGCTCCAAAACCAGAAAAACCAGCTGAA
14566_5#67	AACCAGAGAATCCAGCTCCAAAACCAGAAAAACCAGCTGAA
14566_5#68	AACCAGAGAATCCAGCTCCAAAACCAGAAAAACCAGCTGAA
14566_5#71	AACCAGAGAATCCAGCTCCAAAACCAGAAAAACCAGCTGAA
14566_6#17	AACCAGAGAATCCAGCTCCAAAACCAGAAAAACCAGCTGAA
14566_6#22	AACCAGAGAATCCAGCTCCAAAACCAGAAAAACCAGCTGAA
14566_6#26	AACCAGAGAATCCAGCTCCAAAACCAGAAAAACCAGCTGAA
14566_6#33	AACCAGAGAATCCAGCTCCAAAACCAGAAAAACCAGCTGAA

14566_6#38	AACCAGAGAATCCAGCTCCAAAACCAGAAAAACCAGCTGAA
14566_6#59	AACCAGAGAATCCAGCTCCAAAACCAGAAAAACCAGCTGAA
14566_6#61	AACCAGAGAATCCAGCTCCAAAACCAGAAAAACCAGCTGAA
14566_6#65	AACCAGAGAATCCAGCTCCAAAACCAGAAAAACCAGCTGAA
14566_6#69	AACCAGAGAATCCAGCTCCAAAACCAGAAAAACCAGCTGAA
14913_2#11	AACCAGAGAATCCAGCTCCAAAACCAGAAAAACCAGCTGAA
14913_2#13	AACCAGAGAATCCAGCTCCAAAACCAGAAAAACCAGCTGAA
14913_2#18	AACCAGAGAATCCAGCTCCAAAACCAGAAAAACCAGCTGAA
14913_2#3	AACCAGAGAATCCAGCTCCAAAACCAGAAAAACCAGCTGAA
14913_2#31	AACCAGAGAATCCAGCTCCAAAACCAGAAAAACCAGCTGAA
14913_2#32	AACCAGAGAATCCAGCTCCAAAACCAGAAAAACCAGCTGAA
14913_2#36	AACCAGAGAATCCAGCTCCAAAACCAGAAAAACCAGCTGAA
14913_2#37	AACCAGAGAATCCAGCTCCAAAACCAGAAAAACCAGCTGAA
14913_2#42	AACCAGAGAATCCAGCTCCAAAACCAGAAAAACCAGCTGAA
14913_2#51	AACCAGAGAATCCAGCTCCAAAACCAGAAAAACCAGCTGAA
14913_2#6	AACCAGAGAATCCAGCTCCAAAACCAGAAAAACCAGCTGAA
14913_2#61	AACCAGAGAATCCAGCTCCAAAACCAGAAAAACCAGCTGAA
14913_2#64	AACCAGAGAATCCAGCTCCAAAACCAGAAAAACCAGCTGAA
14913_2#68	AACCAGAGAATCCAGCTCCAAAACCAGAAAAACCAGCTGAA
14913_2#78	AACCAGAGAATCCAGCTCCAAAACCAGAAAAACCAGCTGAA
14913_2#87	AACCAGAGAATCCAGCTCCAAAACCAGAAAAACCAGCTGAA
14913_2#88	AACCAGAGAATCCAGCTCCAAAACCAGAAAAACCAGCTGAA
14913_2#91	AACCAGAGAATCCAGCTCCAAAACCAGAAAAACCAGCTGAA
14913_3#15	AACCAGAGAATCCAGCTCCAAAACCAGAAAAACCAGCTGAA
14913_3#28	AACCAGAGAATCCAGCTCCAAAACCAGAAAAACCAGCTGAA
14913_3#30	AACCAGAGAATCCAGCTCCAAAACCAGAAAAACCAGCTGAA
14913_3#31	AACCAGAGAATCCAGCTCCAAAACCAGAAAAACCAGCTGAA
14913_3#34	AACCAGAGAATCCAGCTCCAAAACCAGAAAAACCAGCTGAA
14913_3#40	AACCAGAGAATCCAGCTCCAAAACCAGAAAAACCAGCTGAA
14913_3#48	AACCAGAGAATCCAGCTCCAAAACCAGAAAAACCAGCTGAA
14913_3#6	AACCAGAGAATCCAGCTCCAAAACCAGAAAAACCAGCTGAA
14913_3#67	AACCAGAGAATCCAGCTCCAAAACCAGAAAAACCAGCTGAA
14913_3#74	AACCAGAGAATCCAGCTCCAAAACCAGAAAAACCAGCTGAA
14913_3#77	AACCAGAGAATCCAGCTCCAAAACCAGAAAAACCAGCTGAA
14913_3#88	AACCAGAGAATCCAGCTCCAAAACCAGAAAAACCAGCTGAA
14913_3#93	AACCAGAGAATCCAGCTCCAAAACCAGAAAAACCAGCTGAA
14913_4#13	AACCAGAGAATCCAGCTCCAAAACCAGAAAAACCAGCTGAA
14913_4#14	AACCAGAGAATCCAGCTCCAAAACCAGAAAAACCAGCTGAA
14913_4#18	AACCAGAGAATCCAGCTCCAAAACCAGAAAAACCAGCTGAA
14913_4#20	AACCAGAGAATCCAGCTCCAAAACCAGAAAAACCAGCTGAA
14913_4#5	AACCAGAGAATCCAGCTCCAAAACCAGAAAAACCAGCTGAA
14913_4#55	AACCAGAGAATCCAGCTCCAAAACCAGAAAAACCAGCTGAA
14913_4#73	AACCAGAGAATCCAGCTCCAAAACCAGAAAAACCAGCTGAA
14913_4#76	AACCAGAGAATCCAGCTCCAAAACCAGAAAAACCAGCTGAA
14913_4#78	AACCAGAGAATCCAGCTCCAAAACCAGAAAAACCAGCTGAA
14913_4#79	AACCAGAGAATCCAGCTCCAAAACCAGAAAAACCAGCTGAA
14913_4#83	AACCAGAGAATCCAGCTCCAAAACCAGAAAAACCAGCTGAA
14913_4#90	AACCAGAGAATCCAGCTCCAAAACCAGAAAAACCAGCTGAA
14913_4#91	AACCAGAGAATCCAGCTCCAAAACCAGAAAAACCAGCTGAA
14913_5#37	AACCAGAGAATCCAGCTCCAAAACCAGAAAAACCAGCTGAA
14913_5#47	AACCAGAGAATCCAGCTCCAAAACCAGAAAAACCAGCTGAA
14913_6#12	AACCAGAGAATCCAGCTCCAAAACCAGAAAAACCAGCTGAA
14913_6#29	AACCAGAGAATCCAGCTCCAAAACCAGAAAAACCAGCTGAA
14913_6#60	AACCAGAGAATCCAGCTCCAAAACCAGAAAAACCAGCTGAA
14913_6#68	AACCAGAGAATCCAGCTCCAAAACCAGAAAAACCAGCTGAA
14913_6#76	AACCAGAGAATCCAGCTCCAAAACCAGAAAAACCAGCTGAA
14913_6#82	AACCAGAGAATCCAGCTCCAAAACCAGAAAAACCAGCTGAA
14913_6#92	AACCAGAGAATCCAGCTCCAAAACCAGAAAAACCAGCTGAA
14913_7#17	AACCAGAGAATCCAGCTCCAAAACCAGAAAAACCAGCTGAA
14913_7#18	AACCAGAGAATCCAGCTCCAAAACCAGAAAAACCAGCTGAA
14913_7#3	AACCAGAGAATCCAGCTCCAAAACCAGAAAAACCAGCTGAA
14913_7#31	AACCAGAGAATCCAGCTCCAAAACCAGAAAAACCAGCTGAA
14913_7#33	AACCAGAGAATCCAGCTCCAAAACCAGAAAAACCAGCTGAA
14913_7#4	AACCAGAGAATCCAGCTCCAAAACCAGAAAAACCAGCTGAA
14913_7#52	AACCAGAGAATCCAGCTCCAAAACCAGAAAAACCAGCTGAA
14913_7#85	AACCAGAGAATCCAGCTCCAAAACCAGAAAAACCAGCTGAA
14913_7#89	AACCAGAGAATCCAGCTCCAAAACCAGAAAAACCAGCTGAA
14913_7#9	AACCAGAGAATCCAGCTCCAAAACCAGAAAAACCAGCTGAA
14936_5#1	AACCAGAGAATCCAGCTCCAAAACCAGAAAAACCAGCTGAA
14936_5#10	AACCAGAGAATCCAGCTCCAAAACCAGAAAAACCAGCTGAA
14936_5#18	AACCAGAGAATCCAGCTCCAAAACCAGAAAAACCAGCTGAA
14936_5#19	AACCAGAGAATCCAGCTCCAAAACCAGAAAAACCAGCTGAA
14936_5#22	AACCAGAGAATCCAGCTCCAAAACCAGAAAAACCAGCTGAA
14936_5#27	AACCAGAGAATCCAGCTCCAAAACCAGAAAAACCAGCTGAA
14936_5#31	AACCAGAGAATCCAGCTCCAAAACCAGAAAAACCAGCTGAA
14936_5#6	AACCAGAGAATCCAGCTCCAAAACCAGAAAAACCAGCTGAA
14936_5#61	AACCAGAGAATCCAGCTCCAAAACCAGAAAAACCAGCTGAA
14936_5#80	AACCAGAGAATCCAGCTCCAAAACCAGAAAAACCAGCTGAA
14936_5#82	AACCAGAGAATCCAGCTCCAAAACCAGAAAAACCAGCTGAA

14936_5#85	AACCAGAGAATCCAGCTCCAAAACCAGAAAAACCAGCTGAA
14936_6#24	AACCAGAGAATCCAGCTCCAAAACCAGAAAAACCAGCTGAA
14936_6#25	AACCAGAGAATCCAGCTCCAAAACCAGAAAAACCAGCTGAA
14936_6#27	AACCAGAGAATCCAGCTCCAAAACCAGAAAAACCAGCTGAA
14936_6#29	AACCAGAGAATCCAGCTCCAAAACCAGAAAAACCAGCTGAA
15378_2#1	AACCAGAGAATCCAGCTCCAAAACCAGAAAAACCAGCTGAA
15378_2#30	AACCAGAGAATCCAGCTCCAAAACCAGAAAAACCAGCTGAA
15378_2#50	AACCAGAGAATCCAGCTCCAAAACCAGAAAAACCAGCTGAA
15378_2#74	AACCAGAGAATCCAGCTCCAAAACCAGAAAAACCAGCTGAA
15378_4#25	AACCAGAGAATCCAGCTCCAAAACCAGAAAAACCAGCTGAA
15378_4#26	AACCAGAGAATCCAGCTCCAAAACCAGAAAAACCAGCTGAA
15531_2#18	AACCAGAGAATCCAGCTCCAAAACCAGAAAAACCAGCTGAA
15531_2#90	AACCAGAGAATCCAGCTCCAAAACCAGAAAAACCAGCTGAA
15531_2#95	AACCAGAGAATCCAGCTCCAAAACCAGAAAAACCAGCTGAA
15531_3#4	AACCAGAGAATCCAGCTCCAAAACCAGAAAAACCAGCTGAA
15531_3#63	AACCAGAGAATCCAGCTCCAAAACCAGAAAAACCAGCTGAA
15531_3#92	AACCAGAGAATCCAGCTCCAAAACCAGAAAAACCAGCTGAA
15531_4#50	AACCAGAGAATCCAGCTCCAAAACCAGAAAAACCAGCTGAA
15531_4#89	AACCAGAGAATCCAGCTCCAAAACCAGAAAAACCAGCTGAA
15531_4#91	AACCAGAGAATCCAGCTCCAAAACCAGAAAAACCAGCTGAA
15531_5#10	AACCAGAGAATCCAGCTCCAAAACCAGAAAAACCAGCTGAA
15531_5#15	AACCAGAGAATCCAGCTCCAAAACCAGAAAAACCAGCTGAA
15531_5#31	AACCAGAGAATCCAGCTCCAAAACCAGAAAAACCAGCTGAA
15531_5#35	AACCAGAGAATCCAGCTCCAAAACCAGAAAAACCAGCTGAA
15531_5#40	AACCAGAGAATCCAGCTCCAAAACCAGAAAAACCAGCTGAA
15531_5#53	AACCAGAGAATCCAGCTCCAAAACCAGAAAAACCAGCTGAA
15531_6#18	AACCAGAGAATCCAGCTCCAAAACCAGAAAAACCAGCTGAA
15531_6#22	AACCAGAGAATCCAGCTCCAAAACCAGAAAAACCAGCTGAA
15531_6#24	AACCAGAGAATCCAGCTCCAAAACCAGAAAAACCAGCTGAA
15531_6#29	AACCAGAGAATCCAGCTCCAAAACCAGAAAAACCAGCTGAA
15531_6#32	AACCAGAGAATCCAGCTCCAAAACCAGAAAAACCAGCTGAA
15531_6#34	AACCAGAGAATCCAGCTCCAAAACCAGAAAAACCAGCTGAA
15531_6#37	AACCAGAGAATCCAGCTCCAAAACCAGAAAAACCAGCTGAA
15531_6#45	AACCAGAGAATCCAGCTCCAAAACCAGAAAAACCAGCTGAA
15531_6#54	AACCAGAGAATCCAGCTCCAAAACCAGAAAAACCAGCTGAA
15531_6#58	AACCAGAGAATCCAGCTCCAAAACCAGAAAAACCAGCTGAA
15531_6#60	AACCAGAGAATCCAGCTCCAAAACCAGAAAAACCAGCTGAA
15531_6#63	AACCAGAGAATCCAGCTCCAAAACCAGAAAAACCAGCTGAA
15531_6#70	AACCAGAGAATCCAGCTCCAAAACCAGAAAAACCAGCTGAA
15873_2#57	AACCAGAGAATCCAGCTCCAAAACCAGAAAAACCAGCTGAA
15873_2#59	AACCAGAGAATCCAGCTCCAAAACCAGAAAAACCAGCTGAA
15873_3#10	AACCAGAGAATCCAGCTCCAAAACCAGAAAAACCAGCTGAA
15873_3#12	AACCAGAGAATCCAGCTCCAAAACCAGAAAAACCAGCTGAA
15873_3#23	AACCAGAGAATCCAGCTCCAAAACCAGAAAAACCAGCTGAA
15873_4#58	AACCAGAGAATCCAGCTCCAAAACCAGAAAAACCAGCTGAA
15873_4#59	AACCAGAGAATCCAGCTCCAAAACCAGAAAAACCAGCTGAA
15873_5#66	AACCAGAGAATCCAGCTCCAAAACCAGAAAAACCAGCTGAA
17138_3#11	AACCAGAGAATCCAGCTCCAAAACCAGAAAAACCAGCTGAA
17138_3#47	AACCAGAGAATCCAGCTCCAAAACCAGAAAAACCAGCTGAA
17138_3#72	AACCAGAGAATCCAGCTCCAAAACCAGAAAAACCAGCTGAA
17150_5#71	AACCAGAGAATCCAGCTCCAAAACCAGAAAAACCAGCTGAA
17150_5#72	AACCAGAGAATCCAGCTCCAAAACCAGAAAAACCAGCTGAA
17150_6#11	AACCAGAGAATCCAGCTCCAAAACCAGAAAAACCAGCTGAA
17150_6#32	AACCAGAGAATCCAGCTCCAAAACCAGAAAAACCAGCTGAA
17150_6#72	AACCAGAGAATCCAGCTCCAAAACCAGAAAAACCAGCTGAA
17175_7#23	AACCAGAGAATCCAGCTCCAAAACCAGAAAAACCAGCTGAA
17175_7#31	AACCAGAGAATCCAGCTCCAAAACCAGAAAAACCAGCTGAA
17175_7#38	AACCAGAGAATCCAGCTCCAAAACCAGAAAAACCAGCTGAA
17175_7#67	AACCAGAGAATCCAGCTCCAAAACCAGAAAAACCAGCTGAA
17175_8#13	AACCAGAGAATCCAGCTCCAAAACCAGAAAAACCAGCTGAA
17175_8#20	AACCAGAGAATCCAGCTCCAAAACCAGAAAAACCAGCTGAA
17175_8#33	AACCAGAGAATCCAGCTCCAAAACCAGAAAAACCAGCTGAA
17175_8#72	AACCAGAGAATCCAGCTCCAAAACCAGAAAAACCAGCTGAA
17428_8#1	AACCAGAGAATCCAGCTCCAAAACCAGAAAAACCAGCTGAA
17428_8#10	AACCAGAGAATCCAGCTCCAAAACCAGAAAAACCAGCTGAA
17428_8#101	AACCAGAGAATCCAGCTCCAAAACCAGAAAAACCAGCTGAA
17428_8#102	AACCAGAGAATCCAGCTCCAAAACCAGAAAAACCAGCTGAA
17428_8#104	AACCAGAGAATCCAGCTCCAAAACCAGAAAAACCAGCTGAA
17428_8#105	AACCAGAGAATCCAGCTCCAAAACCAGAAAAACCAGCTGAA
17428_8#107	AACCAGAGAATCCAGCTCCAAAACCAGAAAAACCAGCTGAA
17428_8#108	AACCAGAGAATCCAGCTCCAAAACCAGAAAAACCAGCTGAA
17428_8#2	AACCAGAGAATCCAGCTCCAAAACCAGAAAAACCAGCTGAA
17428_8#29	AACCAGAGAATCCAGCTCCAAAACCAGAAAAACCAGCTGAA
17428_8#33	AACCAGAGAATCCAGCTCCAAAACCAGAAAAACCAGCTGAA
17428_8#34	AACCAGAGAATCCAGCTCCAAAACCAGAAAAACCAGCTGAA
17428_8#45	AACCAGAGAATCCAGCTCCAAAACCAGAAAAACCAGCTGAA
17428_8#49	AACCAGAGAATCCAGCTCCAAAACCAGAAAAACCAGCTGAA
17428_8#5	AACCAGAGAATCCAGCTCCAAAACCAGAAAAACCAGCTGAA
17428_8#52	AACCAGAGAATCCAGCTCCAAAACCAGAAAAACCAGCTGAA

```
17428_8#57   AACCAGAGAATCCAGCTCCAAAACCAGAAAAACCAGCTGAA
17428_8#58   AACCAGAGAATCCAGCTCCAAAACCAGAAAAACCAGCTGAA
17428_8#60   AACCAGAGAATCCAGCTCCAAAACCAGAAAAACCAGCTGAA
17428_8#61   AACCAGAGAATCCAGCTCCAAAACCAGAAAAACCAGCTGAA
17428_8#76   AACCAGAGAATCCAGCTCCAAAACCAGAAAAACCAGCTGAA
17428_8#81   AACCAGAGAATCCAGCTCCAAAACCAGAAAAACCAGCTGAA
17428_8#82   AACCAGAGAATCCAGCTCCAAAACCAGAAAAACCAGCTGAA
17428_8#9    AACCAGAGAATCCAGCTCCAAAACCAGAAAAACCAGCTGAA
17428_8#90   AACCAGAGAATCCAGCTCCAAAACCAGAAAAACCAGCTGAA
17428_8#91   AACCAGAGAATCCAGCTCCAAAACCAGAAAAACCAGCTGAA
17794_7#81   AACCAGAGAATCCAGCTCCAAAACCAGAAAAACCAGCTGAA
17870_7#1    AACCAGAGAATCCAGCTCCAAAACCAGAAAAACCAGCTGAA
17870_7#115  AACCAGAGAATCCAGCTCCAAAACCAGAAAAACCAGCTGAA
17870_7#118  AACCAGAGAATCCAGCTCCAAAACCAGAAAAACCAGCTGAA
17870_7#184  AACCAGAGAATCCAGCTCCAAAACCAGAAAAACCAGCTGAA
17870_7#187  AACCAGAGAATCCAGCTCCAAAACCAGAAAAACCAGCTGAA
17870_8#10   AACCAGAGAATCCAGCTCCAAAACCAGAAAAACCAGCTGAA
17870_8#102  AACCAGAGAATCCAGCTCCAAAACCAGAAAAACCAGCTGAA
17870_8#107  AACCAGAGAATCCAGCTCCAAAACCAGAAAAACCAGCTGAA
17870_8#108  AACCAGAGAATCCAGCTCCAAAACCAGAAAAACCAGCTGAA
17870_8#118  AACCAGAGAATCCAGCTCCAAAACCAGAAAAACCAGCTGAA
17870_8#120  AACCAGAGAATCCAGCTCCAAAACCAGAAAAACCAGCTGAA
17870_8#137  AACCAGAGAATCCAGCTCCAAAACCAGAAAAACCAGCTGAA
17870_8#145  AACCAGAGAATCCAGCTCCAAAACCAGAAAAACCAGCTGAA
17870_8#146  AACCAGAGAATCCAGCTCCAAAACCAGAAAAACCAGCTGAA
17870_8#149  AACCAGAGAATCCAGCTCCAAAACCAGAAAAACCAGCTGAA
17870_8#154  AACCAGAGAATCCAGCTCCAAAACCAGAAAAACCAGCTGAA
17870_8#156  AACCAGAGAATCCAGCTCCAAAACCAGAAAAACCAGCTGAA
17870_8#163  AACCAGAGAATCCAGCTCCAAAACCAGAAAAACCAGCTGAA
17870_8#20   AACCAGAGAATCCAGCTCCAAAACCAGAAAAACCAGCTGAA
17870_8#23   AACCAGAGAATCCAGCTCCAAAACCAGAAAAACCAGCTGAA
17870_8#26   AACCAGAGAATCCAGCTCCAAAACCAGAAAAACCAGCTGAA
17870_8#27   AACCAGAGAATCCAGCTCCAAAACCAGAAAAACCAGCTGAA
17870_8#44   AACCAGAGAATCCAGCTCCAAAACCAGAAAAACCAGCTGAA
17870_8#45   AACCAGAGAATCCAGCTCCAAAACCAGAAAAACCAGCTGAA
17870_8#46   AACCAGAGAATCCAGCTCCAAAACCAGAAAAACCAGCTGAA
17870_8#56   AACCAGAGAATCCAGCTCCAAAACCAGAAAAACCAGCTGAA
17870_8#63   AACCAGAGAATCCAGCTCCAAAACCAGAAAAACCAGCTGAA
17870_8#70   AACCAGAGAATCCAGCTCCAAAACCAGAAAAACCAGCTGAA
17870_8#72   AACCAGAGAATCCAGCTCCAAAACCAGAAAAACCAGCTGAA
17870_8#73   AACCAGAGAATCCAGCTCCAAAACCAGAAAAACCAGCTGAA
17870_8#75   AACCAGAGAATCCAGCTCCAAAACCAGAAAAACCAGCTGAA
17870_8#79   AACCAGAGAATCCAGCTCCAAAACCAGAAAAACCAGCTGAA
17870_8#81   AACCAGAGAATCCAGCTCCAAAACCAGAAAAACCAGCTGAA
17870_8#82   AACCAGAGAATCCAGCTCCAAAACCAGAAAAACCAGCTGAA
17870_8#83   AACCAGAGAATCCAGCTCCAAAACCAGAAAAACCAGCTGAA
17870_8#85   AACCAGAGAATCCAGCTCCAAAACCAGAAAAACCAGCTGAA
17870_8#90   AACCAGAGAATCCAGCTCCAAAACCAGAAAAACCAGCTGAA
17870_8#99   AACCAGAGAATCCAGCTCCAAAACCAGAAAAACCAGCTGAA
17966_5#101  AACCAGAGAATCCAGCTCCAAAACCAGAAAAACCAGCTGAA
17966_5#108  AACCAGAGAATCCAGCTCCAAAACCAGAAAAACCAGCTGAA
17966_5#110  AACCAGAGAATCCAGCTCCAAAACCAGAAAAACCAGCTGAA
17966_5#112  AACCAGAGAATCCAGCTCCAAAACCAGAAAAACCAGCTGAA
17966_5#113  AACCAGAGAATCCAGCTCCAAAACCAGAAAAACCAGCTGAA
17966_5#29   AACCAGAGAATCCAGCTCCAAAACCAGAAAAACCAGCTGAA
17966_5#39   AACCAGAGAATCCAGCTCCAAAACCAGAAAAACCAGCTGAA
17966_5#53   AACCAGAGAATCCAGCTCCAAAACCAGAAAAACCAGCTGAA
17966_5#58   AACCAGAGAATCCAGCTCCAAAACCAGAAAAACCAGCTGAA
17966_5#71   AACCAGAGAATCCAGCTCCAAAACCAGAAAAACCAGCTGAA
17966_5#76   AACCAGAGAATCCAGCTCCAAAACCAGAAAAACCAGCTGAA
17966_5#80   AACCAGAGAATCCAGCTCCAAAACCAGAAAAACCAGCTGAA
17966_5#84   AACCAGAGAATCCAGCTCCAAAACCAGAAAAACCAGCTGAA
17966_5#85   AACCAGAGAATCCAGCTCCAAAACCAGAAAAACCAGCTGAA
17966_5#88   AACCAGAGAATCCAGCTCCAAAACCAGAAAAACCAGCTGAA
17966_5#89   AACCAGAGAATCCAGCTCCAAAACCAGAAAAACCAGCTGAA
17966_5#91   AACCAGAGAATCCAGCTCCAAAACCAGAAAAACCAGCTGAA
17966_5#93   AACCAGAGAATCCAGCTCCAAAACCAGAAAAACCAGCTGAA
17966_5#95   AACCAGAGAATCCAGCTCCAAAACCAGAAAAACCAGCTGAA
17966_5#97   AACCAGAGAATCCAGCTCCAAAACCAGAAAAACCAGCTGAA
17966_5#98   AACCAGAGAATCCAGCTCCAAAACCAGAAAAACCAGCTGAA
18456_1#101  AACCAGAGAATCCAGCTCCAAAACCAGAAAAACCAGCTGAA
18456_1#109  AACCAGAGAATCCAGCTCCAAAACCAGAAAAACCAGCTGAA
18456_1#115  AACCAGAGAATCCAGCTCCAAAACCAGAAAAACCAGCTGAA
18456_1#119  AACCAGAGAATCCAGCTCCAAAACCAGAAAAACCAGCTGAA
18456_1#120  AACCAGAGAATCCAGCTCCAAAACCAGAAAAACCAGCTGAA
18456_1#128  AACCAGAGAATCCAGCTCCAAAACCAGAAAAACCAGCTGAA
18456_1#164  AACCAGAGAATCCAGCTCCAAAACCAGAAAAACCAGCTGAA
18456_1#165  AACCAGAGAATCCAGCTCCAAAACCAGAAAAACCAGCTGAA
18456_1#166  AACCAGAGAATCCAGCTCCAAAACCAGAAAAACCAGCTGAA
```

18456_1#174	AACCAGAGAATCCAGCTCCAAAACCAGAAAAACCAGCTGAA
18456_1#176	AACCAGAGAATCCAGCTCCAAAACCAGAAAAACCAGCTGAA
18456_1#177	AACCAGAGAATCCAGCTCCAAAACCAGAAAAACCAGCTGAA
18456_1#179	AACCAGAGAATCCAGCTCCAAAACCAGAAAAACCAGCTGAA
18456_1#180	AACCAGAGAATCCAGCTCCAAAACCAGAAAAACCAGCTGAA
19341_1#158	AACCAGAGAATCCAGCTCCAAAACCAGAAAAACCAGCTGAA
19341_1#164	AACCAGAGAATCCAGCTCCAAAACCAGAAAAACCAGCTGAA
19341_1#165	AACCAGAGAATCCAGCTCCAAAACCAGAAAAACCAGCTGAA
19341_2#23	AACCAGAGAATCCAGCTCCAAAACCAGAAAAACCAGCTGAA
19341_2#38	AACCAGAGAATCCAGCTCCAAAACCAGAAAAACCAGCTGAA
19341_2#39	AACCAGAGAATCCAGCTCCAAAACCAGAAAAACCAGCTGAA
19341_2#48	AACCAGAGAATCCAGCTCCAAAACCAGAAAAACCAGCTGAA
19341_2#52	AACCAGAGAATCCAGCTCCAAAACCAGAAAAACCAGCTGAA
19341_2#53	AACCAGAGAATCCAGCTCCAAAACCAGAAAAACCAGCTGAA
19341_2#54	AACCAGAGAATCCAGCTCCAAAACCAGAAAAACCAGCTGAA
19341_2#56	AACCAGAGAATCCAGCTCCAAAACCAGAAAAACCAGCTGAA
20402_4#106	AACCAGAGAATCCAGCTCCAAAACCAGAAAAACCAGCTGAA
21523_1#25	AACCAGAGAATCCAGCTCCAAAACCAGAAAAACCAGCTGAA
21523_1#39	AACCAGAGAATCCAGCTCCAAAACCAGAAAAACCAGCTGAA
21523_1#49	AACCAGAGAATCCAGCTCCAAAACCAGAAAAACCAGCTGAA
21523_1#58	AACCAGAGAATCCAGCTCCAAAACCAGAAAAACCAGCTGAA
21523_1#72	AACCAGAGAATCCAGCTCCAAAACCAGAAAAACCAGCTGAA
21999_8#163	AACCAGAGAATCCAGCTCCAAAACCAGAAAAACCAGCTGAA
21999_8#175	AACCAGAGAATCCAGCTCCAAAACCAGAAAAACCAGCTGAA
22027_1#12	AACCAGAGAATCCAGCTCCAAAACCAGAAAAACCAGCTGAA
22027_1#2	AACCAGAGAATCCAGCTCCAAAACCAGAAAAACCAGCTGAA
22027_1#9	AACCAGAGAATCCAGCTCCAAAACCAGAAAAACCAGCTGAA
3850_1#1	AACCAGAGAATCCAGCTCCAAAACCAGAAAAACCAGCTGAA
4350_6#3	AACCAGAGAATCCAGCTCCAAAACCAGAAAAACCAGCTGAA
4350_6#9	AACCAGAGAATCCAGCTCCAAAACCAGAAAAACCAGCTGAA
4353_1#1	AACCAGAGAATCCAGCTCCAAAACCAGAAAAACCAGCTGAA
4353_1#10	AACCAGAGAATCCAGCTCCAAAACCAGAAAAACCAGCTGAA
4353_2#4	AACCAGAGAATCCAGCTCCAAAACCAGAAAAACCAGCTGAA
4353_2#9	AACCAGAGAATCCAGCTCCAAAACCAGAAAAACCAGCTGAA
4370_1#12	AACCAGAGAATCCAGCTCCAAAACCAGAAAAACCAGCTGAA
4525_1#1	AACCAGAGAATCCAGCTCCAAAACCAGAAAAACCAGCTGAA
4525_1#10	AACCAGAGAATCCAGCTCCAAAACCAGAAAAACCAGCTGAA
4525_1#11	AACCAGAGAATCCAGCTCCAAAACCAGAAAAACCAGCTGAA
4525_1#12	AACCAGAGAATCCAGCTCCAAAACCAGAAAAACCAGCTGAA
4525_1#2	AACCAGAGAATCCAGCTCCAAAACCAGAAAAACCAGCTGAA
4525_1#4	AACCAGAGAATCCAGCTCCAAAACCAGAAAAACCAGCTGAA
4525_1#5	AACCAGAGAATCCAGCTCCAAAACCAGAAAAACCAGCTGAA
4525_1#6	AACCAGAGAATCCAGCTCCAAAACCAGAAAAACCAGCTGAA
4525_1#8	AACCAGAGAATCCAGCTCCAAAACCAGAAAAACCAGCTGAA
4525_1#9	AACCAGAGAATCCAGCTCCAAAACCAGAAAAACCAGCTGAA
4525_2#1	AACCAGAGAATCCAGCTCCAAAACCAGAAAAACCAGCTGAA
4525_2#10	AACCAGAGAATCCAGCTCCAAAACCAGAAAAACCAGCTGAA
4525_2#11	AACCAGAGAATCCAGCTCCAAAACCAGAAAAACCAGCTGAA
4525_2#2	AACCAGAGAATCCAGCTCCAAAACCAGAAAAACCAGCTGAA
4525_2#3	AACCAGAGAATCCAGCTCCAAAACCAGAAAAACCAGCTGAA
4525_2#4	AACCAGAGAATCCAGCTCCAAAACCAGAAAAACCAGCTGAA
4525_2#5	AACCAGAGAATCCAGCTCCAAAACCAGAAAAACCAGCTGAA
4525_2#6	AACCAGAGAATCCAGCTCCAAAACCAGAAAAACCAGCTGAA
4525_2#7	AACCAGAGAATCCAGCTCCAAAACCAGAAAAACCAGCTGAA
4525_2#8	AACCAGAGAATCCAGCTCCAAAACCAGAAAAACCAGCTGAA
4525_2#9	AACCAGAGAATCCAGCTCCAAAACCAGAAAAACCAGCTGAA
4525_3#10	AACCAGAGAATCCAGCTCCAAAACCAGAAAAACCAGCTGAA
4525_3#11	AACCAGAGAATCCAGCTCCAAAACCAGAAAAACCAGCTGAA
4525_3#12	AACCAGAGAATCCAGCTCCAAAACCAGAAAAACCAGCTGAA
4525_3#2	AACCAGAGAATCCAGCTCCAAAACCAGAAAAACCAGCTGAA
4525_3#3	AACCAGAGAATCCAGCTCCAAAACCAGAAAAACCAGCTGAA
4525_3#4	AACCAGAGAATCCAGCTCCAAAACCAGAAAAACCAGCTGAA
4525_3#6	AACCAGAGAATCCAGCTCCAAAACCAGAAAAACCAGCTGAA
4525_3#7	AACCAGAGAATCCAGCTCCAAAACCAGAAAAACCAGCTGAA
4525_3#8	AACCAGAGAATCCAGCTCCAAAACCAGAAAAACCAGCTGAA
4525_3#9	AACCAGAGAATCCAGCTCCAAAACCAGAAAAACCAGCTGAA
4525_5#1	AACCAGAGAATCCAGCTCCAAAACCAGAAAAACCAGCTGAA
4525_5#10	AACCAGAGAATCCAGCTCCAAAACCAGAAAAACCAGCTGAA
4525_5#11	AACCAGAGAATCCAGCTCCAAAACCAGAAAAACCAGCTGAA
4525_5#12	AACCAGAGAATCCAGCTCCAAAACCAGAAAAACCAGCTGAA
4525_5#2	AACCAGAGAATCCAGCTCCAAAACCAGAAAAACCAGCTGAA
4525_5#3	AACCAGAGAATCCAGCTCCAAAACCAGAAAAACCAGCTGAA
4525_5#4	AACCAGAGAATCCAGCTCCAAAACCAGAAAAACCAGCTGAA
4525_5#5	AACCAGAGAATCCAGCTCCAAAACCAGAAAAACCAGCTGAA
4525_5#6	AACCAGAGAATCCAGCTCCAAAACCAGAAAAACCAGCTGAA
4525_5#7	AACCAGAGAATCCAGCTCCAAAACCAGAAAAACCAGCTGAA
4525_5#8	AACCAGAGAATCCAGCTCCAAAACCAGAAAAACCAGCTGAA
4525_5#9	AACCAGAGAATCCAGCTCCAAAACCAGAAAAACCAGCTGAA
4525_6#1	AACCAGAGAATCCAGCTCCAAAACCAGAAAAACCAGCTGAA

4525_6#10	AACCAGAGAATCCAGCTCCAAAACCAGAAAAACCAGCTGAA
4525_6#11	AACCAGAGAATCCAGCTCCAAAACCAGAAAAACCAGCTGAA
4525_6#12	AACCAGAGAATCCAGCTCCAAAACCAGAAAAACCAGCTGAA
4525_6#2	AACCAGAGAATCCAGCTCCAAAACCAGAAAAACCAGCTGAA
4525_6#3	AACCAGAGAATCCAGCTCCAAAACCAGAAAAACCAGCTGAA
4525_6#4	AACCAGAGAATCCAGCTCCAAAACCAGAAAAACCAGCTGAA
4525_6#5	AACCAGAGAATCCAGCTCCAAAACCAGAAAAACCAGCTGAA
4525_6#6	AACCAGAGAATCCAGCTCCAAAACCAGAAAAACCAGCTGAA
4525_6#7	AACCAGAGAATCCAGCTCCAAAACCAGAAAAACCAGCTGAA
4525_6#9	AACCAGAGAATCCAGCTCCAAAACCAGAAAAACCAGCTGAA
4525_7#1	AACCAGAGAATCCAGCTCCAAAACCAGAAAAACCAGCTGAA
4525_7#10	AACCAGAGAATCCAGCTCCAAAACCAGAAAAACCAGCTGAA
4525_7#11	AACCAGAGAATCCAGCTCCAAAACCAGAAAAACCAGCTGAA
4525_7#12	AACCAGAGAATCCAGCTCCAAAACCAGAAAAACCAGCTGAA
4525_7#2	AACCAGAGAATCCAGCTCCAAAACCAGAAAAACCAGCTGAA
4525_7#3	AACCAGAGAATCCAGCTCCAAAACCAGAAAAACCAGCTGAA
4525_7#5	AACCAGAGAATCCAGCTCCAAAACCAGAAAAACCAGCTGAA
4525_7#6	AACCAGAGAATCCAGCTCCAAAACCAGAAAAACCAGCTGAA
4525_7#7	AACCAGAGAATCCAGCTCCAAAACCAGAAAAACCAGCTGAA
4525_7#9	AACCAGAGAATCCAGCTCCAAAACCAGAAAAACCAGCTGAA
4525_8#1	AACCAGAGAATCCAGCTCCAAAACCAGAAAAACCAGCTGAA
4525_8#10	AACCAGAGAATCCAGCTCCAAAACCAGAAAAACCAGCTGAA
4525_8#11	AACCAGAGAATCCAGCTCCAAAACCAGAAAAACCAGCTGAA
4525_8#12	AACCAGAGAATCCAGCTCCAAAACCAGAAAAACCAGCTGAA
4525_8#2	AACCAGAGAATCCAGCTCCAAAACCAGAAAAACCAGCTGAA
4525_8#3	AACCAGAGAATCCAGCTCCAAAACCAGAAAAACCAGCTGAA
4525_8#4	AACCAGAGAATCCAGCTCCAAAACCAGAAAAACCAGCTGAA
4525_8#6	AACCAGAGAATCCAGCTCCAAAACCAGAAAAACCAGCTGAA
4525_8#7	AACCAGAGAATCCAGCTCCAAAACCAGAAAAACCAGCTGAA
4525_8#8	AACCAGAGAATCCAGCTCCAAAACCAGAAAAACCAGCTGAA
4525_8#9	AACCAGAGAATCCAGCTCCAAAACCAGAAAAACCAGCTGAA
4564_1#3	AACCAGAGAATCCAGCTCCAAAACCAGAAAAACCAGCTGAA
4564_1#5	AACCAGAGAATCCAGCTCCAAAACCAGAAAAACCAGCTGAA
4564_1#8	AACCAGAGAATCCAGCTCCAAAACCAGAAAAACCAGCTGAA
4564_2#8	AACCAGAGAATCCAGCTCCAAAACCAGAAAAACCAGCTGAA
4564_2#9	AACCAGAGAATCCAGCTCCAAAACCAGAAAAACCAGCTGAA
4752_1#1	AACCAGAGAATCCAGCTCCAAAACCAGAAAAACCAGCTGAA
4752_1#2	AACCAGAGAATCCAGCTCCAAAACCAGAAAAACCAGCTGAA
4752_1#5	AACCAGAGAATCCAGCTCCAAAACCAGAAAAACCAGCTGAA
4752_1#8	AACCAGAGAATCCAGCTCCAAAACCAGAAAAACCAGCTGAA
4752_1#9	AACCAGAGAATCCAGCTCCAAAACCAGAAAAACCAGCTGAA
4880_1#1	AACCAGAGAATCCAGCTCCAAAACCAGAAAAACCAGCTGAA
4880_1#10	AACCAGAGAATCCAGCTCCAAAACCAGAAAAACCAGCTGAA
4880_1#4	AACCAGAGAATCCAGCTCCAAAACCAGAAAAACCAGCTGAA
4880_1#5	AACCAGAGAATCCAGCTCCAAAACCAGAAAAACCAGCTGAA
4880_1#8	AACCAGAGAATCCAGCTCCAAAACCAGAAAAACCAGCTGAA
4880_1#9	AACCAGAGAATCCAGCTCCAAAACCAGAAAAACCAGCTGAA
5390_2#1	AACCAGAGAATCCAGCTCCAAAACCAGAAAAACCAGCTGAA
5390_2#3	AACCAGAGAATCCAGCTCCAAAACCAGAAAAACCAGCTGAA
5390_3#2	AACCAGAGAATCCAGCTCCAAAACCAGAAAAACCAGCTGAA
5390_5#2	AACCAGAGAATCCAGCTCCAAAACCAGAAAAACCAGCTGAA
5390_6#6	AACCAGAGAATCCAGCTCCAAAACCAGAAAAACCAGCTGAA
5390_6#7	AACCAGAGAATCCAGCTCCAAAACCAGAAAAACCAGCTGAA
5390_7#1	AACCAGAGAATCCAGCTCCAAAACCAGAAAAACCAGCTGAA
5390_7#3	AACCAGAGAATCCAGCTCCAAAACCAGAAAAACCAGCTGAA
5390_7#8	AACCAGAGAATCCAGCTCCAAAACCAGAAAAACCAGCTGAA
5390_8#3	AACCAGAGAATCCAGCTCCAAAACCAGAAAAACCAGCTGAA
6308_7#10	AACCAGAGAATCCAGCTCCAAAACCAGAAAAACCAGCTGAA
6308_7#11	AACCAGAGAATCCAGCTCCAAAACCAGAAAAACCAGCTGAA
6308_7#13	AACCAGAGAATCCAGCTCCAAAACCAGAAAAACCAGCTGAA
6308_7#14	AACCAGAGAATCCAGCTCCAAAACCAGAAAAACCAGCTGAA
6308_7#15	AACCAGAGAATCCAGCTCCAAAACCAGAAAAACCAGCTGAA
6308_7#16	AACCAGAGAATCCAGCTCCAAAACCAGAAAAACCAGCTGAA
6308_7#17	AACCAGAGAATCCAGCTCCAAAACCAGAAAAACCAGCTGAA
6308_7#18	AACCAGAGAATCCAGCTCCAAAACCAGAAAAACCAGCTGAA
6308_7#19	AACCAGAGAATCCAGCTCCAAAACCAGAAAAACCAGCTGAA
6308_7#2	AACCAGAGAATCCAGCTCCAAAACCAGAAAAACCAGCTGAA
6308_7#20	AACCAGAGAATCCAGCTCCAAAACCAGAAAAACCAGCTGAA
6308_7#21	AACCAGAGAATCCAGCTCCAAAACCAGAAAAACCAGCTGAA
6308_7#22	AACCAGAGAATCCAGCTCCAAAACCAGAAAAACCAGCTGAA
6308_7#23	AACCAGAGAATCCAGCTCCAAAACCAGAAAAACCAGCTGAA
6308_7#24	AACCAGAGAATCCAGCTCCAAAACCAGAAAAACCAGCTGAA
6308_7#3	AACCAGAGAATCCAGCTCCAAAACCAGAAAAACCAGCTGAA
6308_7#5	AACCAGAGAATCCAGCTCCAAAACCAGAAAAACCAGCTGAA
6308_7#6	AACCAGAGAATCCAGCTCCAAAACCAGAAAAACCAGCTGAA
6308_7#7	AACCAGAGAATCCAGCTCCAAAACCAGAAAAACCAGCTGAA
6308_7#8	AACCAGAGAATCCAGCTCCAAAACCAGAAAAACCAGCTGAA
6308_7#9	AACCAGAGAATCCAGCTCCAAAACCAGAAAAACCAGCTGAA
6308_8#1	AACCAGAGAATCCAGCTCCAAAACCAGAAAAACCAGCTGAA

```
6309_7#10    AACCAGAGAATCCAGCTCCAAAACCAGAAAAACCAGCTGAA
6309_7#12    AACCAGAGAATCCAGCTCCAAAACCAGAAAAACCAGCTGAA
6309_7#14    AACCAGAGAATCCAGCTCCAAAACCAGAAAAACCAGCTGAA
6309_7#15    AACCAGAGAATCCAGCTCCAAAACCAGAAAAACCAGCTGAA
6309_7#16    AACCAGAGAATCCAGCTCCAAAACCAGAAAAACCAGCTGAA
6309_7#17    AACCAGAGAATCCAGCTCCAAAACCAGAAAAACCAGCTGAA
6309_7#18    AACCAGAGAATCCAGCTCCAAAACCAGAAAAACCAGCTGAA
6309_7#20    AACCAGAGAATCCAGCTCCAAAACCAGAAAAACCAGCTGAA
6309_7#21    AACCAGAGAATCCAGCTCCAAAACCAGAAAAACCAGCTGAA
6309_7#23    AACCAGAGAATCCAGCTCCAAAACCAGAAAAACCAGCTGAA
6309_7#24    AACCAGAGAATCCAGCTCCAAAACCAGAAAAACCAGCTGAA
6309_7#3     AACCAGAGAATCCAGCTCCAAAACCAGAAAAACCAGCTGAA
6309_7#5     AACCAGAGAATCCAGCTCCAAAACCAGAAAAACCAGCTGAA
6309_7#6     AACCAGAGAATCCAGCTCCAAAACCAGAAAAACCAGCTGAA
6309_7#7     AACCAGAGAATCCAGCTCCAAAACCAGAAAAACCAGCTGAA
6309_7#8     AACCAGAGAATCCAGCTCCAAAACCAGAAAAACCAGCTGAA
6309_7#9     AACCAGAGAATCCAGCTCCAAAACCAGAAAAACCAGCTGAA
6570_4#1     AACCAGAGAATCCAGCTCCAAAACCAGAAAAACCAGCTGAA
6570_4#10    AACCAGAGAATCCAGCTCCAAAACCAGAAAAACCAGCTGAA
6570_4#11    AACCAGAGAATCCAGCTCCAAAACCAGAAAAACCAGCTGAA
6570_4#12    AACCAGAGAATCCAGCTCCAAAACCAGAAAAACCAGCTGAA
6570_4#13    AACCAGAGAATCCAGCTCCAAAACCAGAAAAACCAGCTGAA
6570_4#14    AACCAGAGAATCCAGCTCCAAAACCAGAAAAACCAGCTGAA
6570_4#15    AACCAGAGAATCCAGCTCCAAAACCAGAAAAACCAGCTGAA
6570_4#16    AACCAGAGAATCCAGCTCCAAAACCAGAAAAACCAGCTGAA
6570_4#17    AACCAGAGAATCCAGCTCCAAAACCAGAAAAACCAGCTGAA
6570_4#2     AACCAGAGAATCCAGCTCCAAAACCAGAAAAACCAGCTGAA
6570_4#3     AACCAGAGAATCCAGCTCCAAAACCAGAAAAACCAGCTGAA
6570_4#4     AACCAGAGAATCCAGCTCCAAAACCAGAAAAACCAGCTGAA
6570_4#5     AACCAGAGAATCCAGCTCCAAAACCAGAAAAACCAGCTGAA
6570_4#6     AACCAGAGAATCCAGCTCCAAAACCAGAAAAACCAGCTGAA
6570_4#7     AACCAGAGAATCCAGCTCCAAAACCAGAAAAACCAGCTGAA
6570_4#8     AACCAGAGAATCCAGCTCCAAAACCAGAAAAACCAGCTGAA
6570_4#9     AACCAGAGAATCCAGCTCCAAAACCAGAAAAACCAGCTGAA
6851_2#73    AACCAGAGAATCCAGCTCCAAAACCAGAAAAACCAGCTGAA
6851_2#74    AACCAGAGAATCCAGCTCCAAAACCAGAAAAACCAGCTGAA
6851_2#76    AACCAGAGAATCCAGCTCCAAAACCAGAAAAACCAGCTGAA
6851_2#79    AACCAGAGAATCCAGCTCCAAAACCAGAAAAACCAGCTGAA
6851_2#80    AACCAGAGAATCCAGCTCCAAAACCAGAAAAACCAGCTGAA
6851_2#82    AACCAGAGAATCCAGCTCCAAAACCAGAAAAACCAGCTGAA
6851_2#83    AACCAGAGAATCCAGCTCCAAAACCAGAAAAACCAGCTGAA
6851_2#84    AACCAGAGAATCCAGCTCCAAAACCAGAAAAACCAGCTGAA
6851_2#85    AACCAGAGAATCCAGCTCCAAAACCAGAAAAACCAGCTGAA
6851_2#86    AACCAGAGAATCCAGCTCCAAAACCAGAAAAACCAGCTGAA
6851_2#87    AACCAGAGAATCCAGCTCCAAAACCAGAAAAACCAGCTGAA
6851_2#88    AACCAGAGAATCCAGCTCCAAAACCAGAAAAACCAGCTGAA
6851_2#90    AACCAGAGAATCCAGCTCCAAAACCAGAAAAACCAGCTGAA
6851_2#91    AACCAGAGAATCCAGCTCCAAAACCAGAAAAACCAGCTGAA
8728_8#89    AACCAGAGAATCCAGCTCCAAAACCAGAAAAACCAGCTGAA
8728_8#90    AACCAGAGAATCCAGCTCCAAAACCAGAAAAACCAGCTGAA
8728_8#91    AACCAGAGAATCCAGCTCCAAAACCAGAAAAACCAGCTGAA
8728_8#92    AACCAGAGAATCCAGCTCCAAAACCAGAAAAACCAGCTGAA
8728_8#93    AACCAGAGAATCCAGCTCCAAAACCAGAAAAACCAGCTGAA
8728_8#94    AACCAGAGAATCCAGCTCCAAAACCAGAAAAACCAGCTGAA
8728_8#96    AACCAGAGAATCCAGCTCCAAAACCAGAAAAACCAGCTGAA
8786_8#21    AACCAGAGAATCCAGCTCCAAAACCAGAAAAACCAGCTGAA
8786_8#22    AACCAGAGAATCCAGCTCCAAAACCAGAAAAACCAGCTGAA
8786_8#23    AACCAGAGAATCCAGCTCCAAAACCAGAAAAACCAGCTGAA
8786_8#24    AACCAGAGAATCCAGCTCCAAAACCAGAAAAACCAGCTGAA
8786_8#25    AACCAGAGAATCCAGCTCCAAAACCAGAAAAACCAGCTGAA
8786_8#26    AACCAGAGAATCCAGCTCCAAAACCAGAAAAACCAGCTGAA
8786_8#28    AACCAGAGAATCCAGCTCCAAAACCAGAAAAACCAGCTGAA
8786_8#29    AACCAGAGAATCCAGCTCCAAAACCAGAAAAACCAGCTGAA
8786_8#30    AACCAGAGAATCCAGCTCCAAAACCAGAAAAACCAGCTGAA
8786_8#31    AACCAGAGAATCCAGCTCCAAAACCAGAAAAACCAGCTGAA
8786_8#32    AACCAGAGAATCCAGCTCCAAAACCAGAAAAACCAGCTGAA
8786_8#33    AACCAGAGAATCCAGCTCCAAAACCAGAAAAACCAGCTGAA
8786_8#34    AACCAGAGAATCCAGCTCCAAAACCAGAAAAACCAGCTGAA
8786_8#35    AACCAGAGAATCCAGCTCCAAAACCAGAAAAACCAGCTGAA
8786_8#36    AACCAGAGAATCCAGCTCCAAAACCAGAAAAACCAGCTGAA
8786_8#37    AACCAGAGAATCCAGCTCCAAAACCAGAAAAACCAGCTGAA
8786_8#38    AACCAGAGAATCCAGCTCCAAAACCAGAAAAACCAGCTGAA
8786_8#39    AACCAGAGAATCCAGCTCCAAAACCAGAAAAACCAGCTGAA
8786_8#40    AACCAGAGAATCCAGCTCCAAAACCAGAAAAACCAGCTGAA
8786_8#41    AACCAGAGAATCCAGCTCCAAAACCAGAAAAACCAGCTGAA
8786_8#42    AACCAGAGAATCCAGCTCCAAAACCAGAAAAACCAGCTGAA
8786_8#43    AACCAGAGAATCCAGCTCCAAAACCAGAAAAACCAGCTGAA
8786_8#44    AACCAGAGAATCCAGCTCCAAAACCAGAAAAACCAGCTGAA
8786_8#45    AACCAGAGAATCCAGCTCCAAAACCAGAAAAACCAGCTGAA
```

```
8786_8#46    AACCAGAGAATCCAGCTCCAAAACCAGAAAAACCAGCTGAA
8786_8#47    AACCAGAGAATCCAGCTCCAAAACCAGAAAAACCAGCTGAA
8786_8#48    AACCAGAGAATCCAGCTCCAAAACCAGAAAAACCAGCTGAA
8786_8#49    AACCAGAGAATCCAGCTCCAAAACCAGAAAAACCAGCTGAA
8786_8#50    AACCAGAGAATCCAGCTCCAAAACCAGAAAAACCAGCTGAA
8786_8#51    AACCAGAGAATCCAGCTCCAAAACCAGAAAAACCAGCTGAA
8786_8#52    AACCAGAGAATCCAGCTCCAAAACCAGAAAAACCAGCTGAA
8786_8#53    AACCAGAGAATCCAGCTCCAAAACCAGAAAAACCAGCTGAA
8786_8#54    AACCAGAGAATCCAGCTCCAAAACCAGAAAAACCAGCTGAA
8786_8#55    AACCAGAGAATCCAGCTCCAAAACCAGAAAAACCAGCTGAA
8786_8#56    AACCAGAGAATCCAGCTCCAAAACCAGAAAAACCAGCTGAA
8786_8#57    AACCAGAGAATCCAGCTCCAAAACCAGAAAAACCAGCTGAA
8786_8#58    AACCAGAGAATCCAGCTCCAAAACCAGAAAAACCAGCTGAA
8786_8#59    AACCAGAGAATCCAGCTCCAAAACCAGAAAAACCAGCTGAA
8786_8#60    AACCAGAGAATCCAGCTCCAAAACCAGAAAAACCAGCTGAA
8786_8#61    AACCAGAGAATCCAGCTCCAAAACCAGAAAAACCAGCTGAA
8786_8#62    AACCAGAGAATCCAGCTCCAAAACCAGAAAAACCAGCTGAA
8786_8#63    AACCAGAGAATCCAGCTCCAAAACCAGAAAAACCAGCTGAA
9517_3#10    AACCAGAGAATCCAGCTCCAAAACCAGAAAAACCAGCTGAA
9517_3#11    AACCAGAGAATCCAGCTCCAAAACCAGAAAAACCAGCTGAA
9517_3#12    AACCAGAGAATCCAGCTCCAAAACCAGAAAAACCAGCTGAA
9517_3#13    AACCAGAGAATCCAGCTCCAAAACCAGAAAAACCAGCTGAA
9517_3#2     AACCAGAGAATCCAGCTCCAAAACCAGAAAAACCAGCTGAA
9517_3#3     AACCAGAGAATCCAGCTCCAAAACCAGAAAAACCAGCTGAA
9517_3#4     AACCAGAGAATCCAGCTCCAAAACCAGAAAAACCAGCTGAA
9517_3#5     AACCAGAGAATCCAGCTCCAAAACCAGAAAAACCAGCTGAA
9517_3#6     AACCAGAGAATCCAGCTCCAAAACCAGAAAAACCAGCTGAA
9517_3#7     AACCAGAGAATCCAGCTCCAAAACCAGAAAAACCAGCTGAA
9517_3#8     AACCAGAGAATCCAGCTCCAAAACCAGAAAAACCAGCTGAA
9517_3#9     AACCAGAGAATCCAGCTCCAAAACCAGAAAAACCAGCTGAA
9789_2#15    AACCAGAGAATCCAGCTCCAAAACCAGAAAAACCAGCTGAA
9789_2#17    AACCAGAGAATCCAGCTCCAAAACCAGAAAAACCAGCTGAA
9789_2#18    AACCAGAGAATCCAGCTCCAAAACCAGAAAAACCAGCTGAA
9789_2#29    AACCAGAGAATCCAGCTCCAAAACCAGAAAAACCAGCTGAA
9789_2#50    AACCAGAGAATCCAGCTCCAAAACCAGAAAAACCAGCTGAA
9789_2#58    AACCAGAGAATCCAGCTCCAAAACCAGAAAAACCAGCTGAA
9789_2#67    AACCAGAGAATCCAGCTCCAAAACCAGAAAAACCAGCTGAA
9789_2#68    AACCAGAGAATCCAGCTCCAAAACCAGAAAAACCAGCTGAA
9789_2#71    AACCAGAGAATCCAGCTCCAAAACCAGAAAAACCAGCTGAA
9789_2#86    AACCAGAGAATCCAGCTCCAAAACCAGAAAAACCAGCTGAA
9789_3#4     AACCAGAGAATCCAGCTCCAAAACCAGAAAAACCAGCTGAA
14913_6#44   AACCAGAAAAACCAGCTCCAAAACCAGAAAAACCAGCTGAA
17428_8#11   AACCAGAAAAACCAGCTCCAAAACCAGAAAAACCAGCTGAA
17428_8#27   AACCAGAAAAACCAGCTCCAAAACCAGAAAAACCAGCTGAA
17428_8#35   AACCAGAAAAACCAGCTCCAAAACCAGAAAAACCAGCTGAA
17428_8#67   AACCAGAAAAACCAGCTCCAAAACCAGAAAAACCAGCTGAA
17428_8#75   AACCAGAAAAACCAGCTCCAAAACCAGAAAAACCAGCTGAA
17428_8#83   AACCAGAAAAACCAGCTCCAAAACCAGAAAAACCAGCTGAA
17870_8#84   AACCAGAAAAACCAGCTCCAAAACCAGAAAAACCAGCTGAA
4525_7#8     AACCAGAAAAACCAGCTCCAAAACCAGAAAAACCAGCTGAA
5390_6#5     AACCAGAAAAACCAGCTCCAAAACCAGAAAAACCAGCTGAA
```
