# Supplementary material for: Bacterial genome-wide association study of hyper-virulent pneumococcal serotype 1 identifies genetic variation associated with neurotropism
Source: Commun Biol. 2020 Oct 8;3:559. doi: 10.1038/s42003-020-01290-9 (PMC7545184; doi:10.1038/s42003-020-01290-9)
Supplement: Supplementary file 6 — Reporting Summary [file 42003_2020_1290_MOESM6_ESM.pdf]

nature research

Corresponding author(s): Chrispin Chaguza
Last updated by author(s): Sep 6, 2020

Reporting Summary

Nature Research wishes to improve the reproducibility of the work that we publish. This form provides structure for consistency and transparency in reporting. For further information on Nature Research policies, see our Editorial Policies and the Editorial Policy Checklist.

Statistics

For all statistical analyses, confirm that the following items are present in the figure legend, table legend, main text, or Methods section.

n/a	Confirmed	
☐	☒	The exact sample size (*n*) for each experimental group/condition, given as a discrete number and unit of measurement
☐	☒	A statement on whether measurements were taken from distinct samples or whether the same sample was measured repeatedly
☐	☒	The statistical test(s) used AND whether they are one- or two-sided<br>*Only common tests should be described solely by name; describe more complex techniques in the Methods section.*
☐	☒	A description of all covariates tested
☐	☒	A description of any assumptions or corrections, such as tests of normality and adjustment for multiple comparisons
☐	☒	A full description of the statistical parameters including central tendency (e.g. means) or other basic estimates (e.g. regression coefficient) AND variation (e.g. standard deviation) or associated estimates of uncertainty (e.g. confidence intervals)
☐	☒	For null hypothesis testing, the test statistic (e.g. *F*, *t*, *r*) with confidence intervals, effect sizes, degrees of freedom and *P* value noted<br>*Give P values as exact values whenever suitable.*
☒	☐	For Bayesian analysis, information on the choice of priors and Markov chain Monte Carlo settings
☒	☐	For hierarchical and complex designs, identification of the appropriate level for tests and full reporting of outcomes
☐	☒	Estimates of effect sizes (e.g. Cohen's *d*, Pearson's *r*), indicating how they were calculated

Our web collection on statistics for biologists contains articles on many of the points above.

Software and code

Policy information about availability of computer code

Data collection	No software was used for data collection.
Data analysis	ggplot2 v3.1.0 , ggcorrplot v0.1.3, BLAST v2.2.30+, GEMMA v0.98, FaST-LMM v2.07.20140723, Bifrost (no version), DSK v2.3.0, Roary v3.6.1, Prokka v1.11, ACT v9.0.5, R v3.5.3, geosphere v1.5.7, extract_PI_SNPs.py (https://gist.github.com/jasonsahl/9306cd014b63cae12154), fastBAPs v1.0.0, PLINK v1.90b4, VCFtools v0.1.16, Snp-Sites v2.3.2, iTOL v2.0, RAxML v7.0.4, Gubbins v1.4.10, GATK v4.0.3.0, SMALT v0.7.4 (www.sourceforge.net/projects/smalt/), PopPUNK v1.1.7, MLSTcheck v2.0.1510612 and SeroBA v1.0.0, SWISS-MODEL, MUSCLE v3.8.31, DNAPlotter v1.0, Robetta and PyMOL v2.4.0

For manuscripts utilizing custom algorithms or software that are central to the research but not yet described in published literature, software must be made available to editors and reviewers. We strongly encourage code deposition in a community repository (e.g. GitHub). See the Nature Research guidelines for submitting code & software for further information.

Data

Policy information about availability of data

All manuscripts must include a data availability statement. This statement should provide the following information, where applicable:
- Accession codes, unique identifiers, or web links for publicly available datasets
- A list of figures that have associated raw data
- A description of any restrictions on data availability

The sequence data used in this study was deposited in the European Nucleotide Archive (ENA) under the accession numbers and isolate metadata provided in Supplementary Data 1-3. The authors declare that all other data supporting the findings of this study are available within the paper and its Supplementary information files.

Field-specific reporting

Please select the one below that is the best fit for your research. If you are not sure, read the appropriate sections before making your selection.

☒ Life sciences ☐ Behavioural & social sciences ☐ Ecological, evolutionary & environmental sciences

For a reference copy of the document with all sections, see nature.com/documents/nr-reporting-summary-flat.pdf

Life sciences study design

All studies must disclose on these points even when the disclosure is negative.

Sample size	The sample size was based on the availability of pneumococcal serotype 1 samples collected through either different projects or bacterial surveillance programs for invasive diseases in different African countries. However, the samples collected represents the largest collection of whole genome sequenced pneumococcal serotype 1 isolates globally (n=909) especially considering that our study focused on a single serotype only.
Data exclusions	We excluded outbreak and carriage associated strains, and strains with ambiguous isolation source (phenotype).
Replication	None
Randomization	N/A
Blinding	N/A

Reporting for specific materials, systems and methods

We require information from authors about some types of materials, experimental systems and methods used in many studies. Here, indicate whether each material, system or method listed is relevant to your study. If you are not sure if a list item applies to your research, read the appropriate section before selecting a response.

Materials & experimental systems

n/a	Involved in the study
☒	☐ Antibodies
☒	☐ Eukaryotic cell lines
☒	☐ Palaeontology and archaeology
☒	☐ Animals and other organisms
☒	☐ Human research participants
☒	☐ Clinical data
☒	☐ Dual use research of concern

Methods

n/a	Involved in the study
☒	☐ ChIP-seq
☒	☐ Flow cytometry
☒	☐ MRI-based neuroimaging
